# Elevated levels of apolipoprotein C3 impair platelet function and reduce thrombosis

**DOI:** 10.1016/j.jtha.2026.01.004

**Published:** 2026-01-29

**Authors:** Waltraud C. Schrottmaier, Julia B. Kral-Pointner, Marion Mussbacher, Manuel Salzmann, Bernhard Hochreiter, Anita Pirabe, Theresa Reiter, Sigrun Badrnya, Johannes A. Schmid, Ivo Volf, Alice Assinger

**Affiliations:** 1 Department of Vascular Biology and Thrombosis Research, Center of Physiology and Pharmacology, Medical University of Vienna, Vienna, Austria; 2 Department of Pharmacology and Toxicology, University of Graz, Graz, Austria; 3 Department of Internal Medicine II/Cardiology, Medical University of Vienna, Vienna, Austria; 4 Department of Physiology, Center of Physiology and Pharmacology, Medical University of Vienna, Vienna, Austria; 5 Ludwig Boltzmann Institute for Cardiovascular Research, Vienna, Austria

**Keywords:** apolipoprotein C-III, blood platelets, cytoskeleton, platelet aggregation, thrombosis

## Abstract

**Background:**

Apolipoprotein C3 (apoC3) circulates primarily on triglyceride-rich lipoproteins and promotes atherosclerosis by fostering lipidemia and inflammation. Thus, apoC3 represents an important cardiovascular risk factor and is associated with cardiovascular events and mortality. Due to their dual nature as hemostatic and immunomodulatory effector cells, platelets play an important role in the development and progression of atherosclerosis and are responsible for thrombotic/thromboembolic events upon plaque rupture.

**Objectives:**

We aimed to elucidate the impact of apoC3 on prothrombotic platelet functions.

**Methods:**

Platelets were isolated from healthy volunteers and the effect of apoC3 on their activation and aggregation was assessed by flow cytometry, ELISA, and light transmission and multiple electrode aggregometry. Platelet spreading and cytoskeletal remodeling were examined by immunofluorescence microscopy. *In vivo* relevance was confirmed in a murine model of FeCl_3_-induced thrombosis.

**Results:**

ApoC3 strongly reduced platelet aggregation independently of the presence of plasma or other blood cells and impaired both α_IIb_β_3_ activation and degranulation. While agonist-induced calcium mobilization, hyperpolarization, and membrane fluidity were not affected, apoC3 slightly reduced AKT phosphorylation and increased vasodilator-stimulated phosphoprotein (VASP) phosphorylation. Furthermore, apoC3-treated platelets displayed impaired lamellipodia formation, which was accompanied by aberrant actin cytoskeleton remodeling. Finally, transfusion of apoC3-treated platelets into mice delayed thrombus formation *in vivo*.

**Conclusion:**

We identified apoC3 as lipoprotein-derived inhibitor of prothrombotic platelet functions, mediating antiaggregatory effects, likely via modulating AKT and VASP signaling and interfering with actin cytoskeleton remodeling to impair lamellipodia formation. Thus, apoC3 may counterbalance its proatherogenic properties on lipid metabolism and inflammation by dampening thrombotic risk in hyperlipidemia.

## 1 | INTRODUCTION

Atherosclerotic cardiovascular disease (ACVD) is independently predicted by hypertriglyceridemia, a cardinal feature of metabolic syndrome [1–3]. Apolipoprotein C3 (apoC3), a 79-amino acid glycoprotein that resides on the surface of very-low-density lipoprotein (VLDL), low-density lipoprotein (LDL), chylomicrons, and high-density lipoprotein, correlates with plasma triglyceride concentration and represents an important cardiovascular risk factor [4]. Accordingly, apoC3 levels are elevated in conditions that represent major risk factors for coronary heart disease, eg, metabolic syndrome or type 2 diabetes [5,6]. Furthermore, elevated apoC3 concentrations predict progression of angiographically detectable coronary artery disease [7–9] and are associated with increased risk for cardiovascular events and cardiovascular mortality [10,11]. Moreover, concentration of apoC3-containing apoB lipoproteins represents the strongest lipoprotein predictor for the progression of coronary atherosclerosis [12–14] and correlates with the occurrence of inflammatory risk markers [15].

Due to its diverse proatherogenic functions, apoC3 plays a central role in the development and progression of ACVD. By inhibiting several lipoprotein lipases, apoC3 is a key regulator of the metabolism of triglyceride-rich lipoproteins and is thus directly and causally implicated in hypertriglyceridemia [4]. ApoC3 interferes with lipolysis through noncompetitive inhibition of endothelium-bound lipoprotein lipase [16,17], hepatic lipase [18], and phospholipase A_1_ activity [19], thereby reducing lipolysis of triglyceride-rich lipoprotein remnants by the liver [18]. Furthermore, apoC3 (together with other C apolipoproteins) also effectively decreases apoE-mediated binding of VLDL and intermediate-density lipoprotein to the LDL receptor [20] and binding of chylomicrons and VLDL particles to the lipolysis-stimulated receptor [21], thereby interfering with clearance of these lipoproteins and enhancing their susceptibility to oxidative modification, which in turn promotes the inflammatory processes of atherosclerosis.

In addition to its effect on lipid metabolism and clearance, apoC3 also contributes to ACVD by directly fostering inflammation. ApoC3 and apoC3-rich VLDL particles induce monocyte adhesion to endothelial cells, which represents a hallmark in the development of atherosclerosis. ApoC3 also activates endothelial cells of both venous and arterial origin, triggering the release of proinflammatory cyto- and chemokines such as interleukin 6 and interleukin 8 as well as upregulating the expression of adhesion molecules vascular cell adhesion molecule 1 and intercellular cell adhesion molecule 1 [22–24]. Concurrently, apoC3 also activates monocytes, potentially via binding to toll-like receptor (TLR) 2/4 heterodimers, leading to secretion of proinflammatory cytokines and free radicals as well as activation of integrin CD11b, which enables endothelial adhesion [24–26]. These findings are especially interesting in light of reports suggesting that endogenous TLR ligands contribute to atherogenesis [27,28].

Eventually, atherosclerotic vascular lesions become prone to rupture or erosion, triggering thrombotic and thromboembolic events and causing life-threatening ischemic tissue damage. ApoC3 levels may foster a procoagulant microenvironment, as high plasma apoC3 is associated with increased activity of numerous coagulation factors, thrombin generation, and clot formation [11,29,30]. Accordingly, elevated plasma apoC3 is associated with an aggravated risk for myocardial infarction and venous thromboembolism [26,29,31].

Platelets are circulating anucleate blood cells with a central function in hemostasis by facilitating cessation of bleeding, but aberrant platelet activation may lead to pathologic thrombosis. Platelets express numerous receptors and adhesion molecules that enable them to rapidly respond to both hemostatic and immunologic stimuli, including TLR2 and TLR4 [32]. Upon activation, platelets release their granule content, which contains a plethora of effector molecules, ranging from cytokines and chemokines, coagulation factors and fibrinolytic agents, to angiogenesis-regulating proteins [33]. In addition, activated platelets can also readily interact with circulating leukocytes and support their activation, adhesion, and transmigration—processes considered to be critical for development and progression of atherosclerotic vascular disease [34]. However, despite the important role of platelet (dys-) function in ACVD, the potential interactions between apoC3 and platelets remain unknown. Therefore, we investigated the effect of physiologically relevant concentrations of apoC3 on prothrombotic platelet function *in vitro* and *in vivo* using a model of FeCl_3_-induced thrombosis.

## 2 | MATERIAL AND METHODS

### 2.1 | Materials

Reagents, antibodies, and resources are listed in Supplementary Table S1. ApoC3 was isolated from delipidated human VLDL via gel filtration and ion exchange chromatography, yielding a purity of 98% (Academy Bio-Medical Company).

### 2.2 | Study approval

Blood and umbilical cords were obtained from healthy volunteers upon informed consent in accordance with the Human Ethics Committee of the Medical University of Vienna (EK237/2004, EK1548/2020, EK1259/2018), the Ethics Committee of the city of Vienna (EK17–245-VK), and the Declaration of Helsinki. Murine *in vivo* experiments were approved by the Animal Care and Use Committee of the Medical University of Vienna and the Austrian Ministry of Education, Science and Research (GZ 2022–0.792.111) in accordance with Animal Research: Reporting of *In Vivo* Experiments (ARRIVE) guidelines and the EU Directive 2010/63 for the protection of animals used for scientific purposes.

### 2.3 | Lipoprotein isolation

VLDL and LDL were isolated from human plasma and oxidized LDL (oxLDL) prepared as previously described [35,36]. Indicated concentrations refer to protein content as determined by Lowry method.

### 2.4 | Platelet isolation

Blood from volunteers devoid of any medication for 10 days was drawn by antecubital venipuncture into vacutainer tubes containing 3.2% sodium citrate. Platelet-rich plasma (PRP) and buffy coat (interphase) were generated by centrifugation (20 minutes, 120 × *g*). Washed platelets (wPLT) and platelet-poor plasma were obtained by centrifuging PRP in the presence of 0.1 μg/mL prostaglandin I_2_ (PGI_2_) (90 seconds, 3000 × *g*) and resuspending platelets in Tyrode-HEPES (TH) buffer (140 mM NaCl, 3 mM KCl, 1 mM MgCl_2_, 16.63 mM NaHCO_3_, 10 mM HEPES, pH 7.4). Platelet analyses were carried out at room temperature unless mentioned otherwise.

### 2.5 | Platelet aggregation

wPLT, PRP, or hirudin-anticoagulated whole blood was incubated with phosphate-buffered saline (PBS) or apoC3 (12.5–50 μg/mL, 3 minutes) before assessing platelet aggregation at 37 °C by light transmission aggregometry (Platelet Aggregation Profiler-8, möLab) or multiple electrode aggregometry (Multiplate Analyzer, DiaPharma), respectively. In selected experiments, wPLT were pretreated with 400 μg/mL maleylated human serum albumin (mHSA) for 15 minutes before addition of apoC3.

### 2.6 | Platelet activation

Buffy coat or wPLT were incubated with PBS or 25 μg/mL apoC3 for 10 minutes. wPLT were subsequently stimulated with 15 μM adenosine diphosphate (ADP), 3 ng/mL convulxin, or 5 μM thrombin receptor activator peptide 6 (TRAP6) for 10 minutes. Cells were stained with fluorescence-labeled antibodies for 20 minutes (see Supplementary Table S1), fixed in 1% formaldehyde for 10 minutes, and analyzed using a CytoFLEX S (V4-B2-Y4-R3) flow cytometer with CytExpert software (both Beckman Coulter). In selected experiments, wPLT were pretreated for 15 minutes with apyrase (1 U/mL); acetylsalicylic acid (1 mM); RGDS peptide (1 mM); pertussis toxin (200 ng/mL); isotype, anti-TLR2, or anti-TLR4 blocking antibodies (10 μg/mL); mHSA (400 μg/mL); or heparin (0.08–10 U/mL) before addition of apoC3. Platelets were identified by CD61 positivity, and their activation was evaluated via expression of CD62P, CD63, CD40L, and activated integrin α_IIb_β_3_ (PAC-1 binding) quantified as percentage of platelets. Monocytes were identified by CD45 positivity and characteristic side scatter, and their activation was evaluated via expression of activated CD11b and CD62L, evaluated as percentage or mean fluorescence intensity of monocytes, respectively. Platelet-monocyte aggregates were identified as CD61-positive monocytes and evaluated as percentage of monocytes.

### 2.7 | Procoagulant platelet formation

wPLT were incubated with PBS or 25 μg/mL apoC3 for 10 minutes and stimulated with a combination of 15 μM TRAP6 and 120 μg/mL collagen for 10 minutes before labeling for 20 minutes with BV605 anti-CD62P and 1 μg/mL AF647-lactadherin, the latter of which binds to exposed phosphatidylserine. Platelets were fixed and analyzed as described above. CD62P and lactadherin double-positive events were identified as procoagulant platelets and quantified as percentage of platelets.

### 2.8 | Platelet surface receptor expression

PRP was incubated with PBS or 25 μg/mL apoC3 for 10 minutes and stained with fluorescence-labeled antibodies or 20 μg/mL ricinus communis agglutinin for 20 minutes (see Supplementary Table S1). Platelets were fixed and analyzed as described above. Platelets were identified by expression of CD61, CD41, or CD42b, and receptor expression was quantified as mean fluorescence intensity of platelets.

### 2.9 | Platelet string formation

Human umbilical vein endothelial cells (HUVECs) were confluently seeded in μ-slides VI^0.4^ (ibidi) and stimulated with 100 ng/mL tumor necrosis factor-α (30 minutes, 37 °C). PRP was stained with CellMask orange (1:1000, 10 minutes) and washed with 0.1 μg/mL PGI_2_ (90 seconds, 3000 × *g*), and platelets were resuspended in perfusion buffer (TH buffer supplemented with 1 mM CaCl_2_, 0.5% bovine serum albumin, and 1 μg/mL fibrinogen). Platelets were incubated with PBS or 25 μg/mL apoC3 for 10 minutes and perfused over HUVECs at microcapillary shear (50 s^−1^, 4 minutes). Platelet string formation on von Willebrand factor (VWF) was visualized by live-cell fluorescence microscopy (30 images/min).

### 2.10 | Platelet-released products

wPLT were incubated with PBS or 25 μg/mL apoC3 for 10 minutes and stimulated for 10 minutes, as described above. Platelets were pelleted in the presence of 0.1 μg/mL PGI_2_ (90 seconds, 3000 × *g*), and the supernatant was cleared of residual platelets (1 minute, 14 000 × *g*). Platelet-derived CXCL4, soluble CD40L, and thrombospondin 1 were quantified by ELISA (R&D Systems) according to manufacturer’s instructions. Adenosine triphosphate was quantified by incubating samples with rLuciferase/Luciferin Reagent and luminescence measured immediately using IVIS Lumina Series III (Caliper LifeSciences).

### 2.11 | Western blotting

wPLT were incubated with PBS or 25 μg/mL apoC3 for 5 minutes, stimulated with 3 μM ADP for 5 minutes, and lysed and denatured in reducing Laemmli buffer (5 minutes, 95 °C). Proteins were separated by sodium dodecyl sulfate-polyacrylamide gel electrophoresis (12% polyacrylamide gels) and blotted onto polyvinylidene fluoride membranes. Membranes were blocked with 4% milk for 60 minutes, probed with primary antibodies overnight, and incubated with appropriate secondary antibodies for 2 hours before visualization using SuperSignal West Femto Maximum Sensitivity Substrate (Thermo Fisher Scientific). Chemiluminescence was measured by FluorChem HD2 Chemiluminescence Imager and AlphaEase FC software (both Alpha Innotech), and relative band intensities were quantified by ImageJ (W. Rasband and contributors, National Institutes of Health).

### 2.12 | Calcium flux

PRP was stained with 3.34 μg/mL Fluo-4 and 6.67 μg/mL FuraRed for 20 minutes, incubated with PBS or 25 μg/mL apoC3 for 5 minutes, and diluted with PBS. Agonist-induced changes in fluorescence were monitored using BD FACS Calibur with CellQuest Pro software (both BD Biosciences). Averaged time series data of Fluo-4/FuraRed ratios (FL-1/FL-3) were generated in FlowJo software and evaluated in GraphPad Prism.

### 2.13 | Vasodilator-stimulated phosphoprotein phosphorylation

PRP was incubated with PBS, apoC3, or positive control PGI_2_ for 3 minutes, fixed in 1% formaldehyde for 10 minutes, and permeabilized in permeabilization buffer for 10 minutes. Platelets were incubated with anti-phospho-vasodilator–stimulated phosphoprotein (VASP) (60 minutes), washed with PBS, centrifuged 10 minutes (1500 × *g*) and stained with flurescein-isothiocyanate (FITC)-anti-mouse antibody (30 minutes). Samples were analyzed using an Accuri C6 flow cytometer with C6 Sampler software (both BD Biosciences) or CytoFLEX S flow cytometer with CytExpert software.

### 2.14 | Membrane fluidity

PRP was stained with CellMask orange (1:1000, 10 minutes), washed with 0.1 μg/mL PGI_2_, centrifuged (90 seconds, 3000 × *g*), and diluted 1:10 in PBS before incubation with PBS or 25 μg/mL apoC3 (10min) and seeding into μ-slides VI^0.4^ coated with fibronectin (20 μg/mL). Membrane fluidity was examined by fluorescence recovery after photobleaching using confocal immunofluorescence microscopy (Nikon A1 plus confocal laser-scanning microscope, Plan Apo 60× objective with numeric aperture = 1.4, 561 nm excitation laser). Briefly, a spherical region of 2 μm diameter was selected within a settled platelet and bleached with strong illumination for 1 second. The recovery of fluorescence within the region was recorded by capturing 1 image/second for 20 seconds and analyzed in ImageJ.

### 2.15 | Membrane potential

wPLT were stained with 10 μM DiBAC_4_(3) for 15 minutes, incubated with PBS or 25 μg/mL apoC3 for 5 minutes, and diluted. Fluorescence was monitored using a BD FACS Calibur. Averaged FL-1 time series were generated in FlowJo and evaluated in GraphPad Prism.

### 2.16 | Platelet settlement

Platelets were stained with CellMask orange and allowed to settle on fibronectin as described above. Settlement was monitored over 65 minutes by taking duplicate images every 4 minutes using confocal immunofluorescence microscopy. Kinetics of platelet settlement were analyzed using a custom macro in ImageJ to segment individual platelets and determine area and coverage.

### 2.17 | Immunofluorescence staining of cytoskeleton

wPLT were incubated with PBS or 25 μg/mL apoC3 for 10 minutes and allowed to adhere to μ-slides VI^0.4^ coated with collagen (100 μg/mL) for 60 minutes before fixation in 1% formaldehyde. Cells were permeabilized (0.5% Triton-X100; 4 minutes), blocked (10% fetal bovine serum, 1.5% bovine serum albumin, 0.005% Triton X-100; 30 minutes), and stained overnight with anti-α/β tubulin (1:100). Cells were then incubated with anti-rabbit-DyLight650 (1:100) and phalloidin-iFluor555 (1:500) for 2 hours before mounting in 83% glycerol. Super-resolution images (3–4/sample) were acquired with Andor iXon Life EMCCD camera and super-resolution radial fluctuations algorithm [37]. Actin cytoskeleton network complexity was quantified by Image J 1.54p using the “AnalyzeSkeleton” plug-in. Briefly, actin images were skeletonized and branch length analyzed without pruning.

### 2.18 | Mice

Wildtype C57BL/6 mice were bred at the Center of Physiology and Pharmacology, Medical University of Vienna, under specific pathogen-free conditions with 12 hour/12 hour light/dark cycle and *ad libitum* access to food and water. Both sexes were used for experiments. Animals were sacrificed by cervical dislocation.

### 2.19 | Murine platelet isolation

Mice (25–35 weeks old) were anesthetized with isoflurane (Forane; Baxter Healthcare Corporation). Blood was drawn from the retro-orbital plexus via heparin-coated microhematocrit tubes, anti-coagulated with 10% acid citrate-dextrose and 25 U/mL heparin, and the mice were subsequently euthanized. A 500-μL sample of blood was mixed with 350 μL TH buffer and centrifuged (5 minutes, 200 × *g*). Platelets and the upper two-thirds of the erythrocyte fraction were transferred into a fresh tube. After another centrifugation (6 minutes, 100 × *g*), the platelet-rich upper fraction was collected. To increase yield, the erythrocyte-rich lower fraction was again mixed with 350 μL TH buffer and centrifuged (6 minutes, 100 × *g*), and the supernatant platelets were collected. Platelets were pelleted (2 minutes, 1000 × *g*) in the presence of 1:25 acid citrate-dextrose and 0.5 U/mL apyrase and resuspended in TH buffer to obtain murine wPLT. Platelet analyses were carried out at room temperature unless mentioned otherwise.

### 2.20 | Murine platelet activation

Murine wPLT were incubated with PBS or 25 μg/mL apoC3 for 10 minutes before stimulation with 25 μM protease-activated receptor 4-activating peptide for 15 minutes, followed by staining with fluorescence-labeled antibodies for 20 minutes (see Supplementary Table S1). Platelets were fixed and analyzed as described above. Platelets were identified by characteristic forward and side scatter, and expression of CD62P or activated α_IIb_β_3_ (JON/A binding) was quantified as percentage of platelets.

### 2.21 | Murine platelet aggregation

To mimic transfusion conditions *in vitro* and investigate effects of partial platelet treatment, we designated murine platelet isolates as “recipient” and “donor” samples. Upon final pelleting, recipient platelets were resuspended at 350 000 cells/μL. Donor platelets were resuspended at 1 400 000 cells/μL and incubated with PBS or 25 μg/mL apoC3 for 10 minutes before dilution with TH buffer to 350 000 cells/μL. Recipient samples were spiked with 10% to 30% treated donor platelets, and collagen-induced aggregation was assessed by light transmission aggregometry.

### 2.22 | *In vivo* thrombosis

Donor platelets were isolated from naïve mice as described above with an additional 0.1 μg/mL PGI_2_ added during final pelleting to allow concentration in smaller volumes for injection. Platelets were labeled with anti-GPIbβ (X488; 1:200) and incubated with PBS or 25 μg/mL apoC3 for 10 minutes. Five-week-old recipient mice were intravenously injected with 5 μL anti-GPIbβ (X488; 0.5 μg/mouse) 1 hour before being transfused with 6.9 × 10^8^ treated donor plate-lets and subjected to FeCl_3_-induced thrombosis of the mesenteric arterioles. Briefly, mice were anesthetized by intraperitoneal injection of ketamine (100 μg/g, Ketasol) and xylazine (10 μg/g, Xylasol), and the small intestines were exteriorized. Thrombosis in mesenteric arterioles was induced by topical application of a drop of 1M FeCl_3_ using filter paper. Thrombus formation was monitored for 40 minutes by intravital microscopy (Olympus IX71 microscope, Visitron Systems GmbH), and time to vessel occlusion was quantified.

### 2.23 | Statistics

Data visualization and statistical evaluation was done in GraphPad Prism. Data were tested for Gaussian distribution by Shapiro–Wilk test. *In vitro* experiments were analyzed as paired datasets by matching samples within individual blood donors. Differences between 2 groups were evaluated by paired *t*-test, differences between multiple groups by Friedman test or mixed-effects analysis in case of missing values. Data with 2 parameters were analyzed by two-way analysis of variance. Normalized changes relative to baseline were evaluated by one-sample *t*-test. Calcium flux and membrane potential in response to different agonists were summarized in 1 graph but analyzed using multiple *t*-tests. Half maximal inhibitory concentrations (IC_50_) were determined by fitting 4 parameter nonlinear regression curves. *P* < .05 was considered statistically significant.

## 3 | RESULTS

### 3.1 | ApoC3 impairs platelet aggregation

**FIGURE 1:**
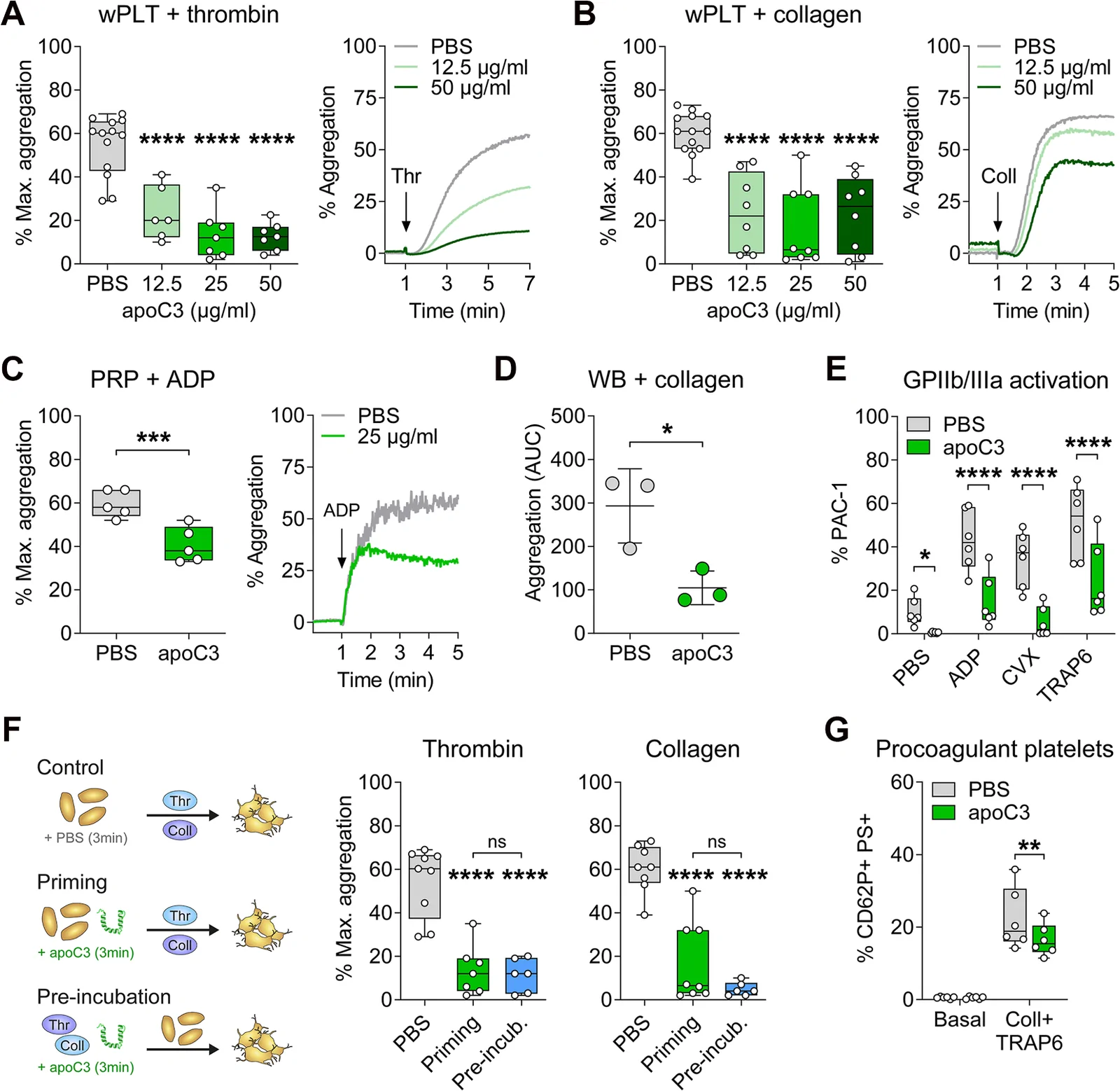
Apolipoprotein C3 (apoC3) impairs platelet aggregation. (A–B) Washed platelets (wPLT) were incubated with phosphate-buffered saline (PBS) or apoC3 (12.5–50 μg/mL, 3 minutes) before stimulation with (A) 35 mU/mL thrombin (Thr) or (B) 5 μg/mL collagen (Coll). Platelet aggregation was monitored by light transmission aggregometry (LTA). Representative curves are shown. *n* = 6–13. (C) Platelet-rich plasma (PRP) was incubated with PBS or 25 μg/mL apoC3 for 3 minutes and stimulated with 5 μM ADP. Platelet aggregation was monitored using LTA. Representative curves are shown. *n* = 5. (D) Whole blood (WB) was incubated with PBS or 25 μg/mL apoC3 for 3 minutes and stimulated with 3 μg/mL collagen. Platelet aggregation was monitored using multiple electrode aggregometry. *n* = 3. (E) wPLT were primed with PBS or 25 μg/mL apoC3 for 10 minutes before stimulation with 15 μM ADP, 3 ng/mL convulxin (CVX), or 5 μM thrombin receptor activator peptide 6 (TRAP6) for 10 minutes. α_IIb_β_3_ activation (PAC-1 antibody binding) was quantified by flow cytometry. *n* = 6. (F) wPLT were incubated for 3 minutes with PBS or primed with 25 μg/mL apoC3 before stimulation with 35 mU/mL thrombin (Thr) or 5 μg/mL collagen (Coll). Alternatively, agonists were preincubated with apoC3 for 3 minutes before stimulation of wPLT. Platelet aggregation was monitored by LTA. *n* = 6–9. (G) wPLT were incubated with PBS or 25 μg/mL apoC3 for 10 minutes before stimulation with 15 μM TRAP6 and 120 μg/mL collagen for 10 minutes. CD62P and phosphatidylserine (PS; lactadherin-binding) double-positive procoagulant platelets were quantified by flow cytometry. *n* = 6. *P < .05, **P < .01, ***P < .001, ****P < .0001. ns: not significant.

When investigating the effect of apoC3 on platelet aggregation, we found that priming of isolated platelets with apoC3 significantly impaired their aggregation in response to different prothrombotic agonists, with low levels of 12.5 to 25 μg/mL apoC3 being sufficient for maximal inhibition of aggregation (Figure 1A, B). This effect remained unaltered in the presence of plasma (Figure 1C) or other blood cells (Figure 1D). In line with this finding, activation of integrin α_IIb_β_3_ (GPIIb/IIIa) was also significantly reduced in apoC3-treated platelets (Figure 1E). Of note, neutralization of ADP and thromboxane A_2_ feedback via apyrase and acetylsalicylic acid, respectively, did not interfere with the inhibitory effect of apoC3 on platelet activation (Supplementary Figure S1). As thrombin was previously shown to cleave apoC3 within its central helix 3 region into 2 fragments (amino acids 1–40 and 41–79) [38,39], we wondered whether preincubating apoC3 with the agonists before adding it to platelets would affect its effect on aggregation. However, neither thrombin nor collagen preincubation of apoC3 affected its antiaggregatory effect (Figure 1F). Furthermore, apoC3 diminished phosphatidylserine exposure on activated platelets (Figure 1G), suggesting that apoC3 may also dampen coagulation.

Of note, neither basal desialylation nor expression levels of key hemostatic and immune receptors, including α_IIb_ (GPIIb) and β_3_ (GPIIIa), were regulated by apoC3 exposure (Supplementary Figure S2), underlining that effects on α_IIb_β_3_ activation are not due to reduced receptor availability.

Beyond classic prothrombotic stimuli, apoC3 also impaired platelet aggregation and α_IIb_β_3_ activation in response to proatherogenic oxLDL (Supplementary Figure S3). As apoC3 circulates primarily on VLDL particles and chylomicrons, we also investigated the effect of VLDL on platelet function. Similar to apoC3, VLDL reduced platelet aggregation, particularly at hypercholesteremic levels (Supplementary Figure S4A–C), as well as α_IIb_β_3_ activation and degranulation (Supplementary Figure S4D, E).

### 3.2 | ApoC3 does not affect platelet binding to VWF

**FIGURE 2:**
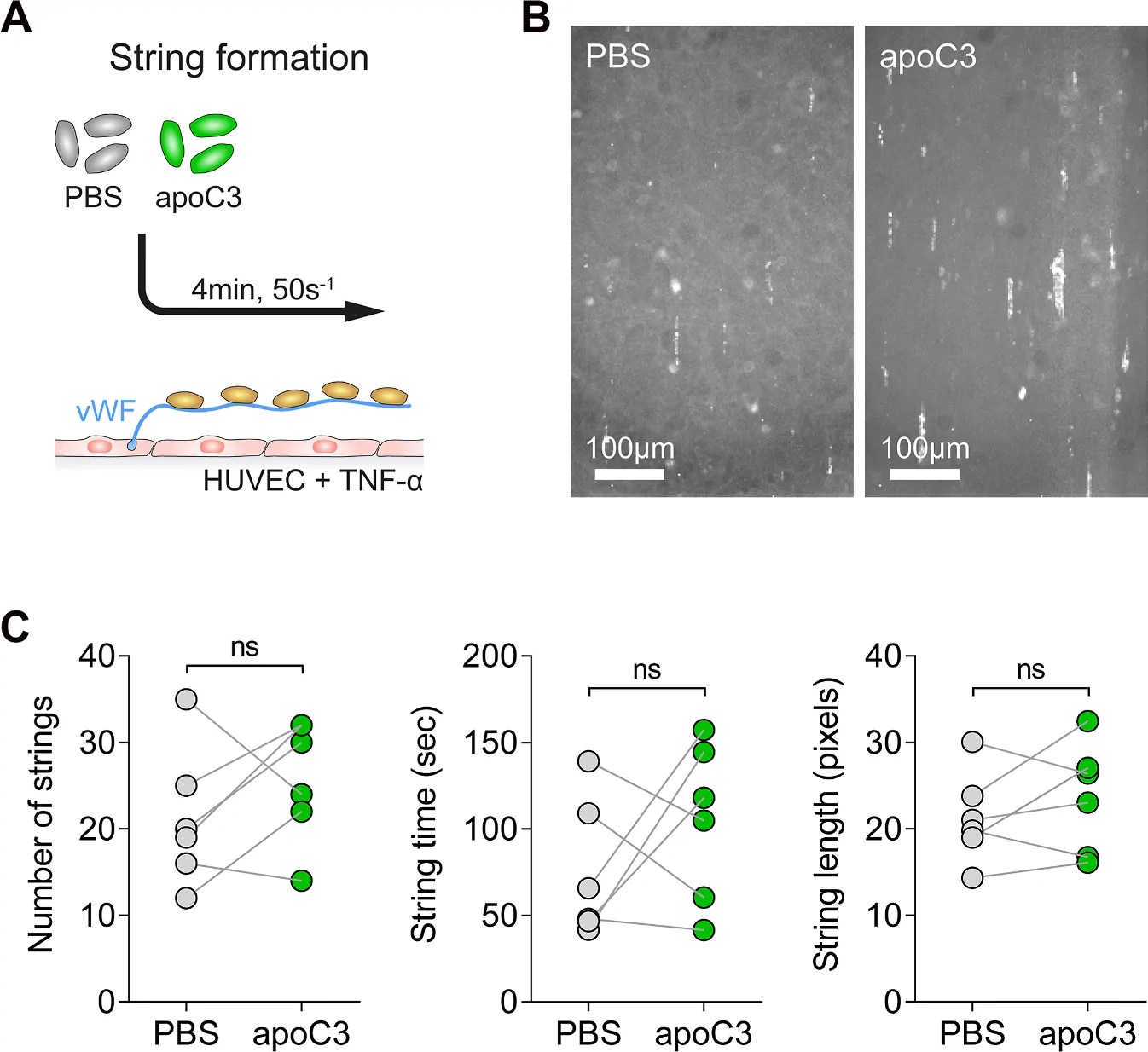
Apolipoprotein C3 (apoC3) does not affect platelet binding to von Willebrand factor (VWF). (A) Washed platelets labeled with CellMask orange were incubated with phosphate-buffered saline (PBS) or 25 μg/mL apoC3 for 10 minutes and perfused under microcapillary shear (50 s^−1^, 4 minutes) over a confluent layer of human umbilical vein endothelial cells (HUVEC) stimulated with 100 ng/mL tumor necrosis factor-α for 30 minutes. Fluorescence images were taken every 2 seconds. Beads-on-a-string–like complexes representing platelets bound to exocytosed unfurled VWF molecules were quantified. (B) Representative fields of view showing cumulative platelet strings via maximal intensity projection. (C) Quantification of number (left), mean duration (middle), and mean length (right) of individual strings per platelet donor. *n* = 6. ns, not significant.

Proinflammatory disease settings such as atherosclerosis foster thrombotic risk in the absence of vascular injury by promoting interaction of platelets with activated endothelium. *In vitro*, platelets firmly attach to VWF strings that originate from activated endothelial cells, forming beads-on-a-string–like complexes that may facilitate leukocyte recruitment and further augment inflammation [40]. Thus, we tested the effect of apoC3 on platelet string formation by perfusing apoC3-treated platelets over activated HUVECs (Figure 2A). However, neither the number of platelet strings nor their duration or length were affected by apoC3 (Figure 2B, C), indicating that apoC3 does not interfere with platelet-VWF binding.

### 3.3 | ApoC3 reduces platelet degranulation

**FIGURE 3:**
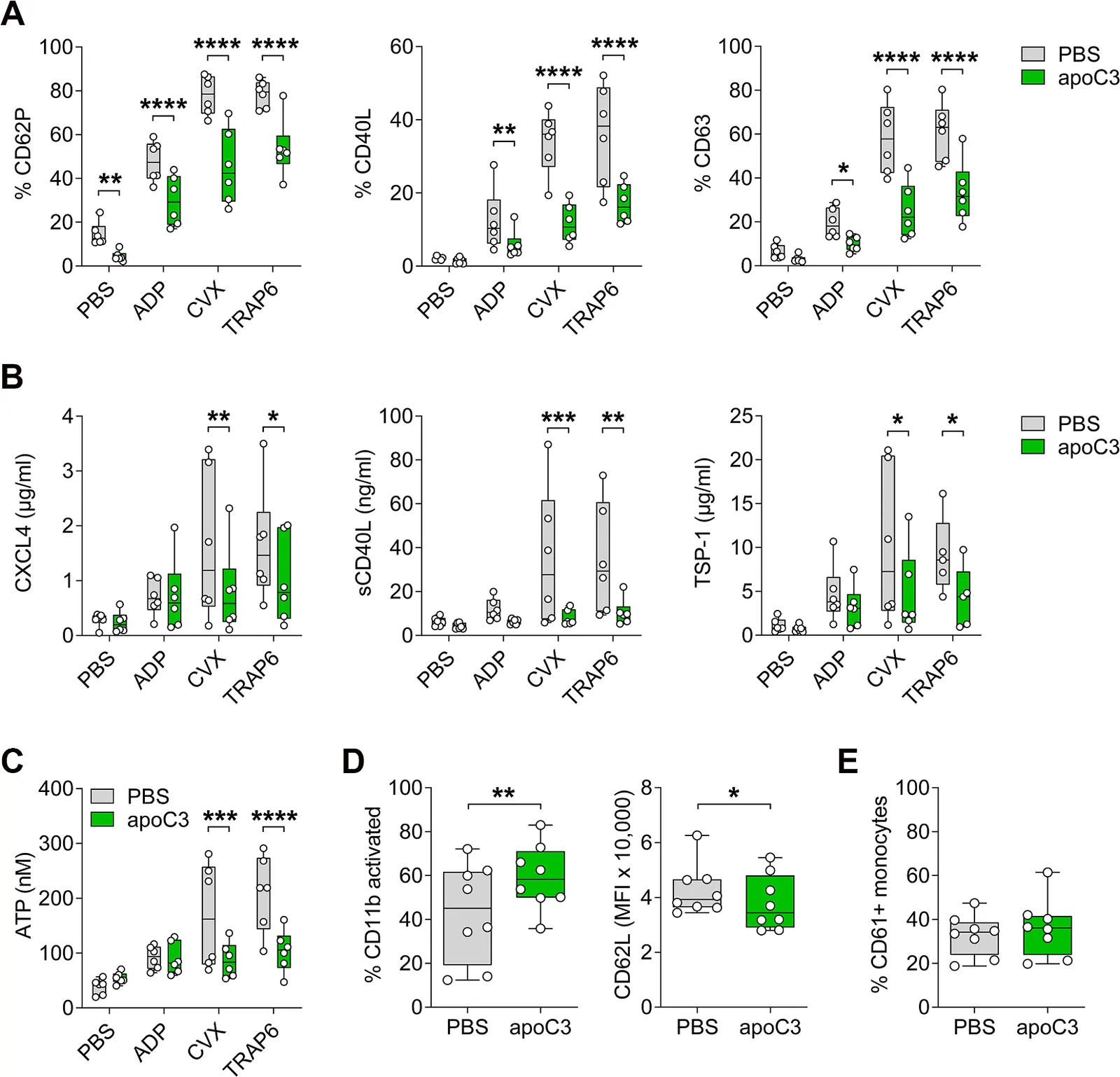
Apolipoprotein C3 (apoC3) reduces platelet degranulation. (A) Washed platelets were primed with phosphate-buffered saline (PBS) or 25 μg/mL apoC3 for 10 minutes before stimulation with 15 μM ADP, 3 ng/mL convulxin (CVX), or 5 μM thrombin receptor activator peptide 6 (TRAP6) for 10 minutes. Surface CD62P (left), CD40L (middle), and CD63 (right) were quantified by flow cytometry. *n* = 6. (B–C) Washed platelets were primed and stimulated as above. Supernatant levels of (B) α-granule-derived CXCL4 (left), soluble CD40L (middle), or thrombospondin 1 (TSP-1) (right) were quantified by ELISA, and (C) dense granule-derived adenosine triphosphate (ATP) was quantified by luciferase assay. *n* = 6. (D–E) Buffy coat was incubated with PBS or 25 μg/mL apoC3 for 10 minutes before flow cytometric quantification of (D) monocyte expression of activated CD11b (left) and CD62L (right) and (E) platelet-monocyte aggregates (CD61+ monocytes). *n* = 8, *P < .05, **P < .01, ***P < .001, ****P < .0001. MFI, mean fluorescence intensity.

Next, we investigated the effect of apoC3 on platelet degranulation. ApoC3 priming of isolated platelets dose-dependently impaired CD62P expression, with IC_50_ values between 25 and 30 μg/mL (Supplementary Figure S5A). Notably, apoC3-mediated platelet inhibition was time-independent with comparable inhibition between 1 and 20 minutes of apoC3 priming (Supplementary Figure S5B). In addition to CD62P, apoC3 also significantly reduced surface levels of CD40L and CD63 (Figure 3A). We also detected decreased levels of α-granule-derived CXCL4, soluble CD40L, and thrombospondin 1 (Figure 3B) as well as dense granule-derived adenosine triphosphate (Figure 3C) in the supernatant of apoC3-treated platelets, underlining that apoC3 forestalls degranulation of both α- and dense granules.

In contrast, apoC3 activated monocytes as evidenced by augmented CD11b activation and reduced surface CD62L, pointing toward CD62L shedding (Figure 3D). However, apoC3 did not alter platelet-monocyte aggregate formation (Figure 3E), which might reflect the opposing effects of apoC3 on these cells.

### 3.4 | ApoC3 modulates platelet membrane potential

**FIGURE 4:**
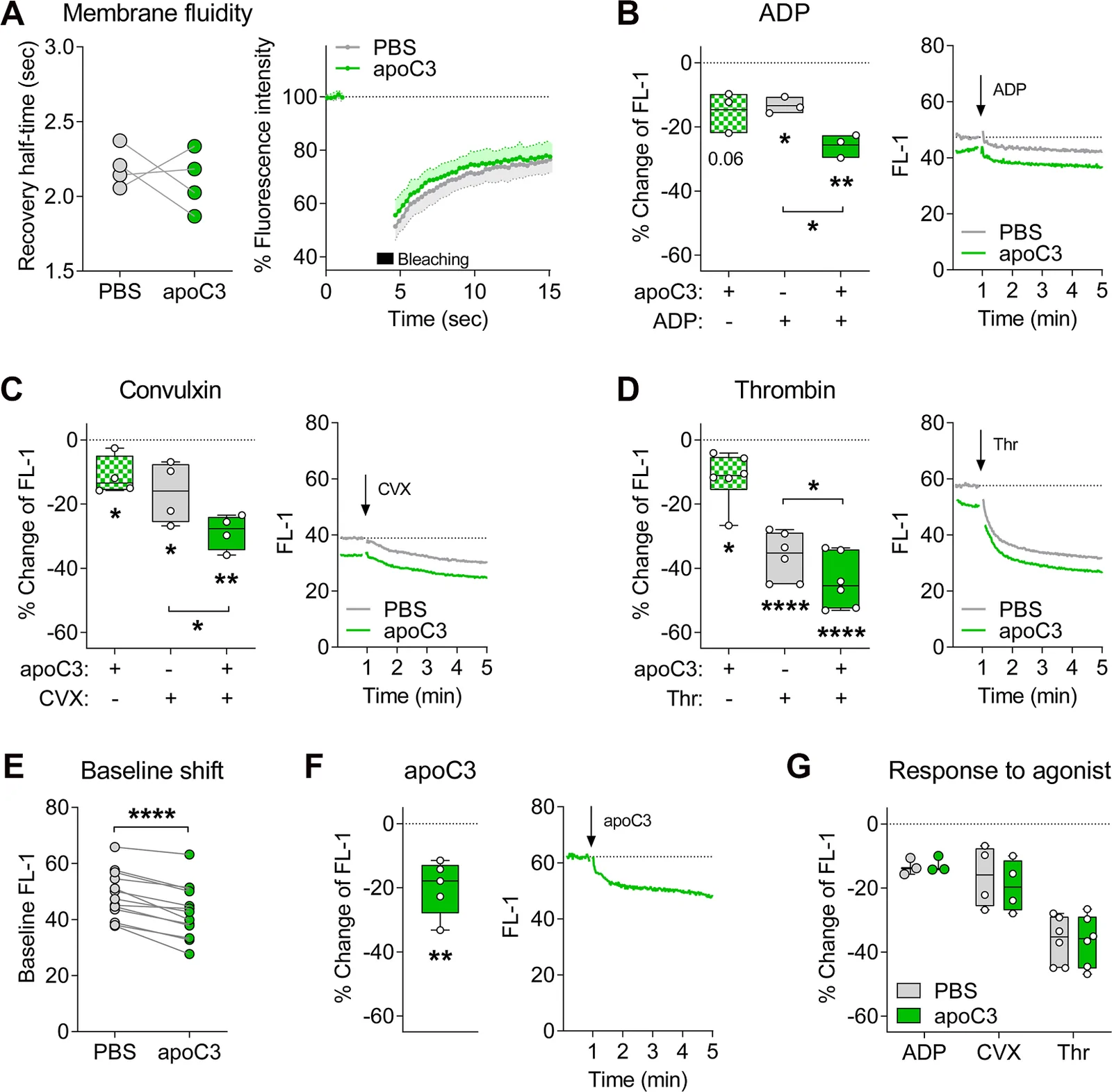
Apolipoprotein C3 (apoC3) modulates platelet membrane potential. (A) Washed platelets labeled with CellMask orange were primed with phosphate-buffered saline (PBS) or 25 μg/mL apoC3 for 10 minutes and allowed to adhere to fibronectin-coated slides for 60 minutes. Membrane fluidity was assessed by fluorescence recovery after photobleaching and quantified as mean half-time of fluorescence recovery. Representative curve shows fluorescence intensity mean and SD derived from all acquired platelets of one donor. *n* = 4. (B–E) Washed platelets were stained with DiBAC_4_(3) for 15 minutes, primed with PBS, or 25 μg/mL apoC3 for 5 minutes, and stimulated with (B) 3 μM ADP, (C) 6 ng/mL convulxin (CVX) or (D) 10 mU/mL thrombin (Thr). Membrane potential was monitored by flow cytometry as changes in fluorescence relative to control (PBS) at baseline. Representative curves are shown. *n* = 3–6. (E) Absolute baseline fluorescence of PBS- and apoC3-primed platelets. *n* = 13. (F) Washed platelets were stained as in (B–E), and relative change in fluorescence upon addition of apoC3 (25 μg/mL) was monitored. Representative curve is shown. *n* = 5. (G) To filter agonist responses, data of (B–D) were normalized by calculating agonist-induced fluorescence changes relative to respective curve baselines for each treatment. *P < .05, **P < .01, ****P < .0001. FL-1, fluorescence channel 1.

ApoC3 incorporates into the phospholipid surface layer of lipid vesicles, but its capacity to bind significantly larger cellular structures is incompletely understood; hence, we assessed whether apoC3 affected physiologic functions of the platelet cell membrane. First, we tested the effect of apoC3 on membrane fluidity, which was unaffected in apoC3-treated platelets as demonstrated by unaltered fluorescence recovery of the stained membrane after photobleaching (Figure 4A). Next, we investigated how apoC3 affected platelet membrane potential by staining them with the electro-sensitive dye DiBAC_4_(3) and monitoring fluorescence upon stimulation. Activation of platelets with ADP, convulxin, or thrombin led to significant hyperpolarization as indicated by a decrease in fluorescence, with apoC3-primed platelets displaying significantly lower membrane potential than control platelets after stimulation (Figure 4B–D). However, curves of apoC3-primed platelets consistently started at lower signal intensities, showing a significant shift in baseline fluorescence (Figure 4E), which suggests that apoC3 itself already affected membrane potential. Indeed, addition of apoC3 to naïve stained platelets resulted in a significant reduction in fluorescence (Figure 4F). When we normalized agonist-induced changes in membrane potential to the respective baseline signal, we could no longer detect any differences between PBS- and apoC3-treated platelets (Figure 4G). This indicates that apoC3 already hyperpolarizes platelets at resting state but does not affect agonist-evoked changes in membrane potential.

### 3.5 | ApoC3-induced platelet inhibition involves AKT and VASP signaling

**FIGURE 5:**
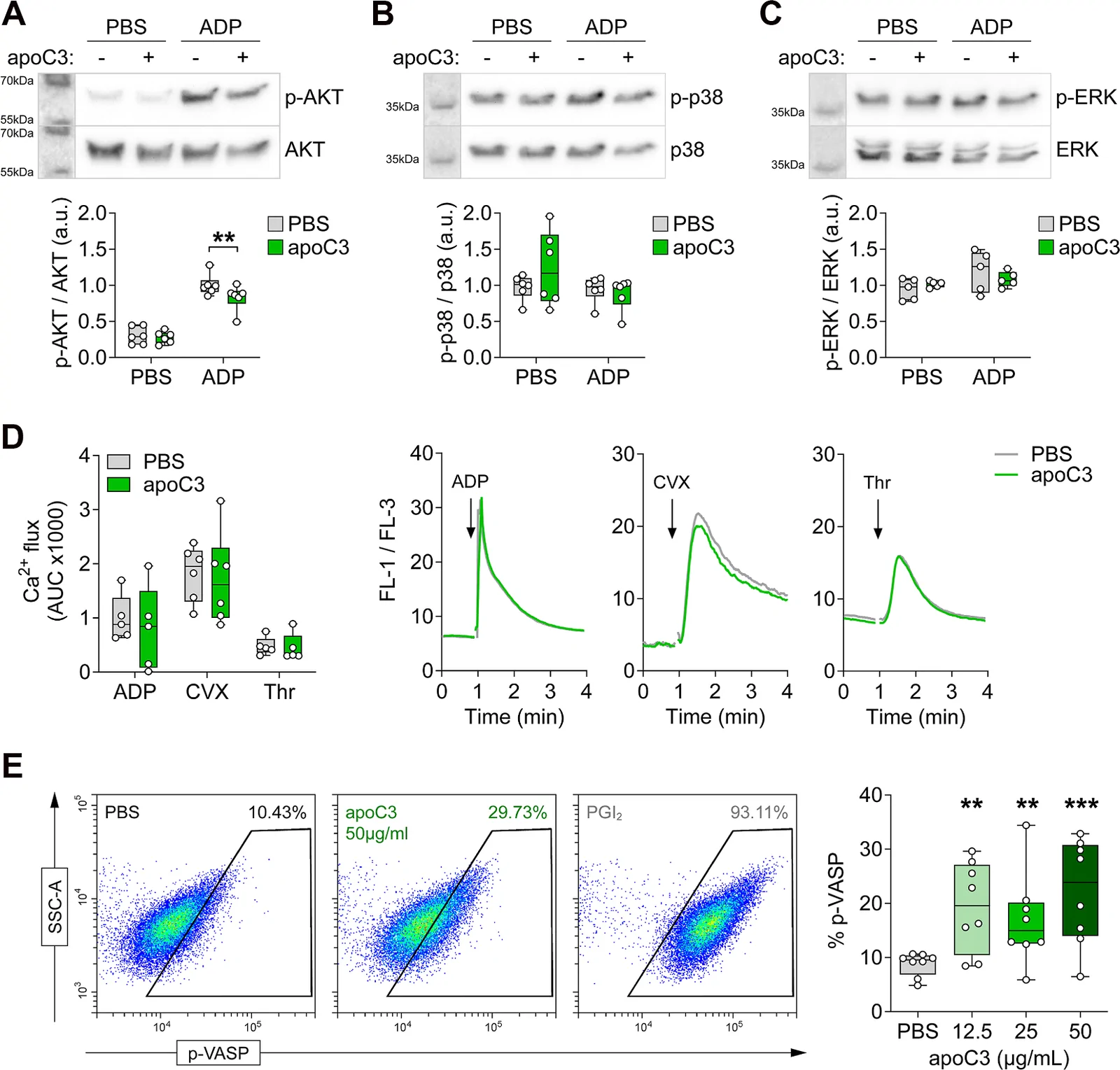
Apolipoprotein C3 (apoC3)-induced platelet inhibition involves AKT and vasodilator-stimulated phosphoprotein (VASP) signaling. (A–C) Washed platelets were primed with phosphate-buffered saline (PBS) or 25 μg/mL apoC3 for 5 minutes before stimulation with 3 μM ADP (5 minutes). Platelet lysates were examined by Western blotting for phosphorylated (p-) and total (A) AKT, (B) p38, and (C) extracellular signal-regulated kinase (ERK). Representative blots and densitometric quantification are shown. *n* = 5–6. (D) Platelet-rich plasma (PRP) was stained with Fluo-4 and FuraRed, primed with PBS or 25 μg/mL apoC3 (5 minutes), and stimulated with 1 μM ADP, 1 ng/mL convulxin (CVX), or 7 mU/mL thrombin (Thr). Intracellular calcium mobilization was monitored by flow cytometry as ratio Fluo-4/FuraRed (FL-1/FL-3). Quantification or area under the curve (AUC) and representative curves are shown. *n* = 5–6. (E) PRP was incubated for 3 minutes with PBS, apoC3 (12.5–50 μg/mL), or 2.5 μg/mL prostaglandin I_2_ (PGI_2_; positive control), and phosphorylation of VASP was quantified by flow cytometry. *n* = 8. Representative plots are shown. *P < .05, **P < .01, ***P < 0.001. a.u., arbitrary units; FL-1, fluorescence channel 1; SSC-A, side scatter area.

To elucidate intracellular signaling events involved in apoC3-mediated platelet inhibition, we analyzed major signaling pathways of platelet activation. We found that priming of platelets with apoC3 led to slightly but significantly reduced phosphorylation of the central signaling hub AKT (Figure 5A), indicating decreased signaling via the phosphatidylinositol 3-kinase (PI3K) pathway. In contrast, phosphorylation of the mitogen-activated protein kinases p38 or extracellular signal-regulated kinase was not regulated by apoC3 (Figure 5B, C). PI3K in platelets is also involved in phospholipase C activation [41] and thus regulates intracellular calcium mobilization. However, we did not detect any effect of apoC3 on calcium flux (Figure 5D). In contrast, apoC3 mildly elevated VASP phosphorylation (Figure 5E), which is associated with α_IIb_β_3_ inhibition [42]. These data suggest that multiple small interferences within the complex signaling network of platelets, including impaired PI3K/AKT signaling and augmented VASP phosphorylation, may synergize to mediate apoC3-induced platelet inhibition.

### 3.6 | ApoC3-induced platelet inhibition is mediated by RGD-containing integrins

To further delineate the molecular mechanisms underlying platelet inhibition by apoC3, we endeavored to characterize apoC3-platelet interactions in more detail. Previously, G_i/o_-coupled receptors, TLR2/4, scavenger receptor SR-B1, and the proteoglycans biglycan and heparan sulfate proteoglycans have been implicated in apoC3 recognition and subsequent cellular responses, with studies focusing on monocytes/macrophages and/or endothelial cells [26,43–46]. While little is known about apolipoprotein receptors on platelets, VLDL engages platelet scavenger receptor CD36 [47], and apoA4 was recently shown to convey antithrombotic effects via α_IIb_β_3_ [48].

**FIGURE 6:**
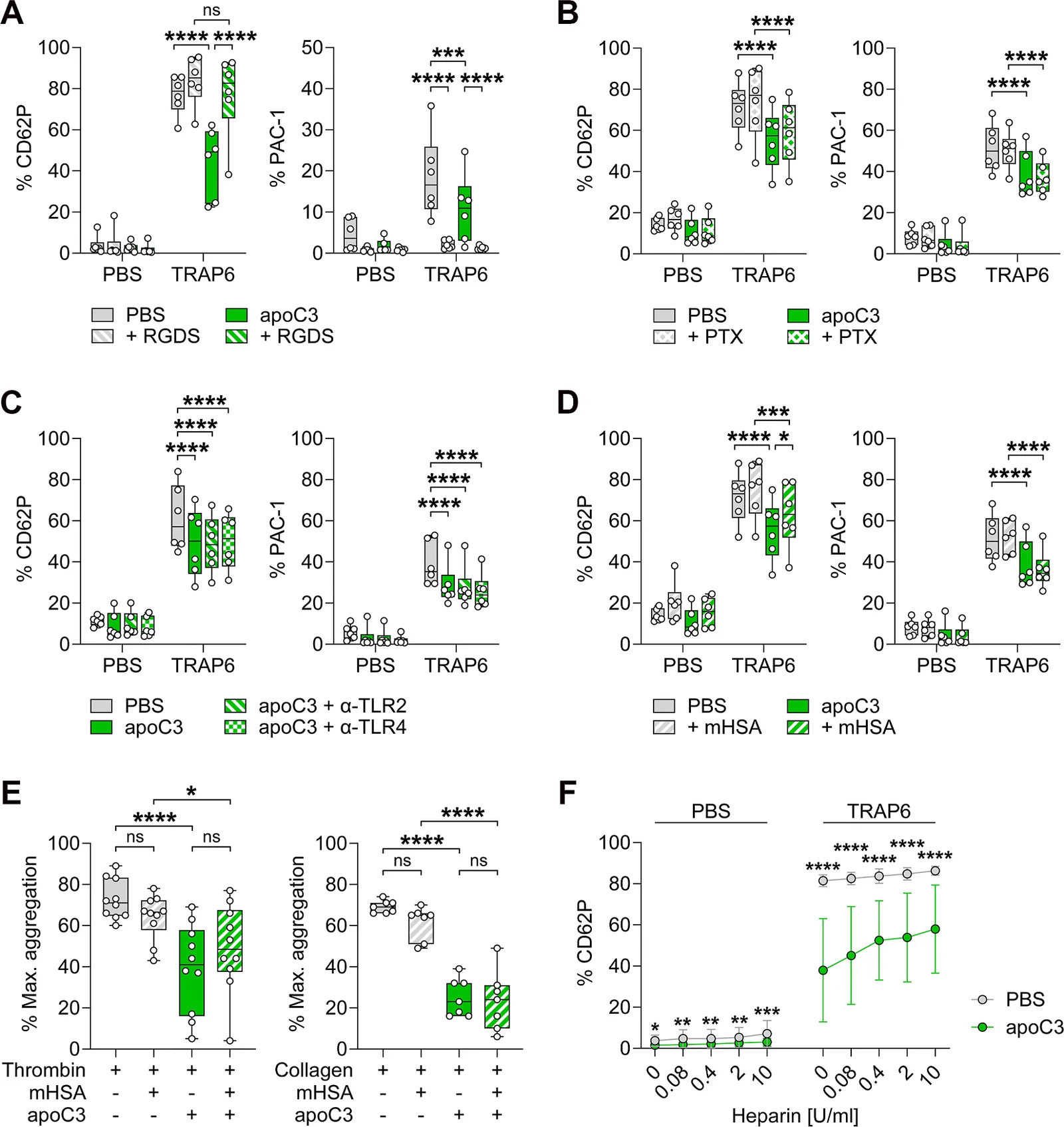
Apolipoprotein C3 (apoC3)-induced platelet inhibition is mediated by RGD-containing integrins and may involve scavenger receptors. (A–D) Washed platelets were pretreated for 15 minutes with (A) RGDS (1 mM), (B) pertussis toxin (PTX; 200 ng/mL), (C) anti-TLR2, anti-TLR4, or isotype control (10 μg/mL), or (D) maleylated human serum albumin (mHSA; 400 μg/mL) before priming for 10 minutes with phosphate-buffered saline (PBS) or 25 μg/mL apoC3 and stimulation with 5 μM thrombin receptor activator peptide 6 (TRAP6) for 10 minutes. CD62P and α_IIb_β_3_ activation (PAC-1 antibody binding) were quantified by flow cytometry. *n* = 5–6. (E) Platelets were pretreated with mHSA (400 μg/ml, 15 minutes) and primed with PBS or 25 μg/mL apoC3 (3 minutes). Platelet aggregation upon addition of 35 mU/mL thrombin or 5 μg/mL collagen was monitored by light transmission aggregometry. *n* = 7–10. (F) Platelets were pretreated with heparin (0.08–10 U/mL, 15 minutes), primed with PBS or 25 μg/mL apoC3 (10 minutes), and stimulated with 5 μM TRAP6 (10 minutes) before quantification of CD62P. Graph shows mean ± SD of *n* = 4. *P < .05, **P < .01, ***P < .001, ****P < .0001. ns, not significant; TLR, toll-like receptor.

To examine the role of α_IIb_β_3_ in apoC3-platelet interactions, we preincubated platelets with RGDS, which competes for integrin’s RGD binding domain and abrogates PAC-1 binding accordingly. While RGDS did not affect TRAP6-induced degranulation of control platelets, it led to a complete rescue of CD62P expression in apoC3-treated platelets (Figure 6A). These results highlight the importance of α_IIb_β_3_ for apoC3-platelet interactions, although it should be noted that integrin α_5_β_1_ recognizes RGD motifs as well [49]. In contrast, neither inhibition of G_i/o_ proteins by pertussis toxin nor blocking of TLR2 or TLR4 modulated apoC3-induced platelet inhibition (Figure 6B, C). Competitive inhibition of scavenger receptors with mHSA also failed to dampen apoC3 effects on PAC-1 binding and aggregation, while mHSA partially rescued TRAP6-induced degranulation in apoC3-treated platelets (Figure 6D, E). Finally, preincubation of platelets with heparin, a soluble competitor for proteoglycans, also failed to prevent CD62P inhibition by apoC3 (Figure 6F).

Our results thus demonstrate that while scavenger receptors may play a minor role in apoC3-platelet interactions, apoC3-mediated platelet dysfunction pivotally depends on RGDS-recognizing integrins α_IIb_β_3_ and/or α_5_β_1_.

### 3.7 | ApoC3 prevents lamellipodia formation by disrupting actin cytoskeletal remodeling

**FIGURE 7:**
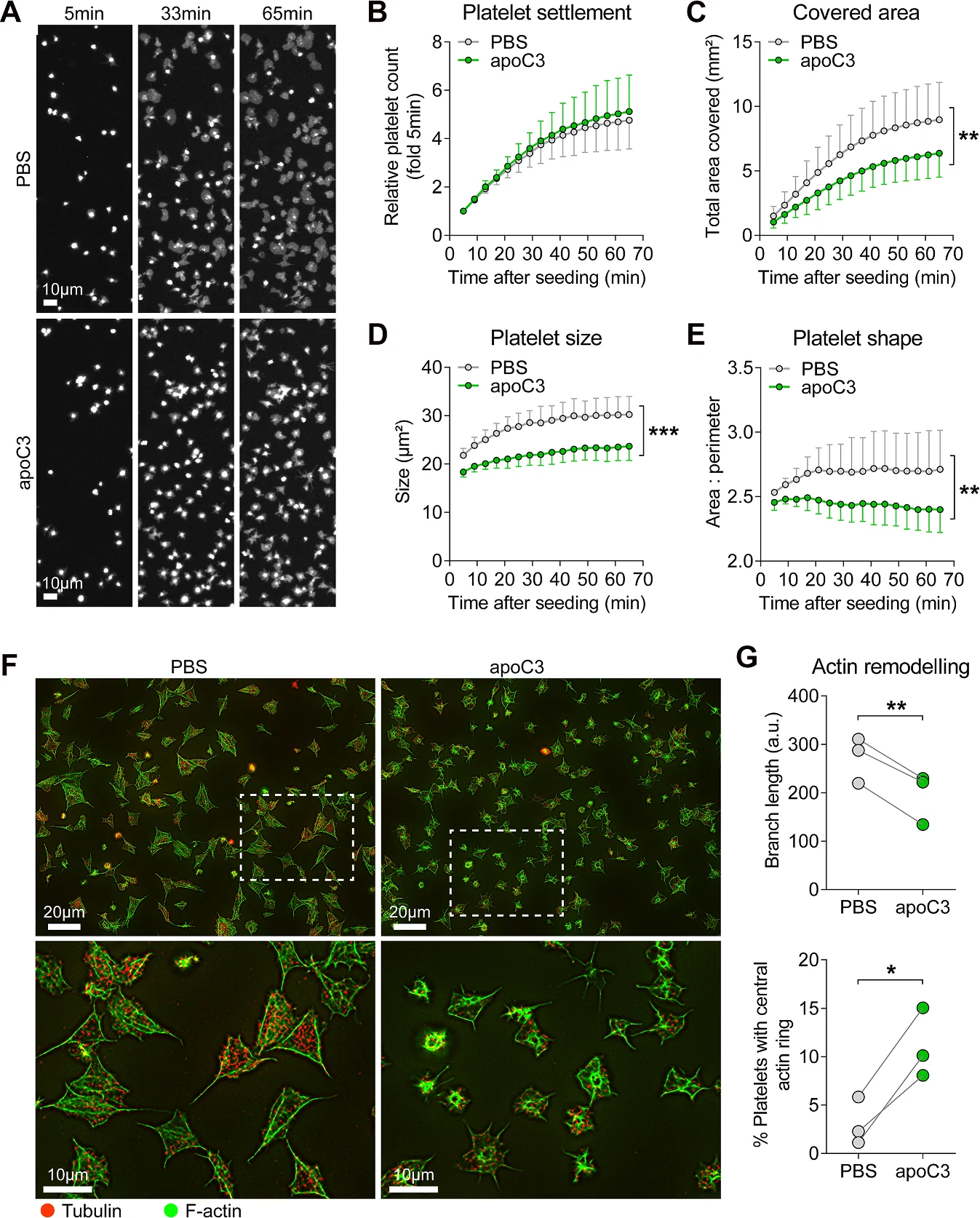
Apolipoprotein C3 (apoC3) prevents lamellipodia formation by disrupting actin cytoskeleton remodeling. (A–E) Washed platelets labeled with CellMask orange were primed with phosphate-buffered saline (PBS) or 25 μg/mL apoC3 for 10 minutes and seeded onto fibronectin-coated slides. Settlement was monitored for 60 minutes after seeding by immunofluorescence microscopy. (A) Representative images taken 5, 33, or 65 minutes after seeding. Images were analyzed for (B) platelet count normalized to first image (5 minutes), (C) total area covered by platelets, (D) mean platelet size, and (E) mean area:perimeter ratio of platelets. *n* = 6. (F–G) Washed platelets were primed with PBS or 25 μg/mL apoC3 (10 minutes) and allowed to adhere to collagen-coated slides for 60 minutes. Fixed slides were stained for filamentous actin (green) and α/β-tubulin (red) and imaged by super-resolution fluorescence microscopy. (F) Representative images. (G) Actin remodeling was quantified as mean length of actin branches (top) and fraction of platelets containing a central actin ring (bottom). *n* = 3. *P < .05, **P < .01, ***P < .001.

Next, we monitored the effect of apoC3 on platelet shape change during settlement and adhesion onto fibrinogen-coated slides. ApoC3-primed platelets appeared to remain more star-shaped, readily forming filopodia but not lamellipodia upon adhesion (Figure 7A, Supplementary Video S1). Indeed, quantification revealed that despite comparable platelet numbers and kinetics of settlement (Figure 7B), apoC3-primed platelets covered significantly less area than control platelets (Figure 7C). Accordingly, individual platelet size was significantly reduced upon apoC3 priming (Figure 7D), which was accompanied by significantly lower area:perimeter ratio as a marker for “roundness” (Figure 7E). To investigate the effect of apoC3 on platelet shape change in more detail, we examined adherent platelets for differences in cytoskeletal structure using super-resolution microscopy and found profound differences of the actin cytoskeleton between PBS- and apoC3-treated platelets (Figure 7F). Adherent control platelets displayed a complex network of filamentous actin, giving it a polygon-like appearance. In contrast, the actin cytoskeleton of apoC3-primed platelets appeared almost wheel-like with numerous filopodia spikes and a central actin ring. Accordingly, image analysis revealed reduced mean branch length of actin fibers and significantly increased the percentage of platelets with a central actin ring upon apoC3 treatment (Figure 7G). Thus, apoC3 causes a profound deficiency in actin cytoskeleton rearrangement, which prevents platelets from spreading fully by diminishing lamellipodia formation.

### 3.8 | Treatment of platelets with apoC3 delays thrombus formation in mice

**FIGURE 8:**
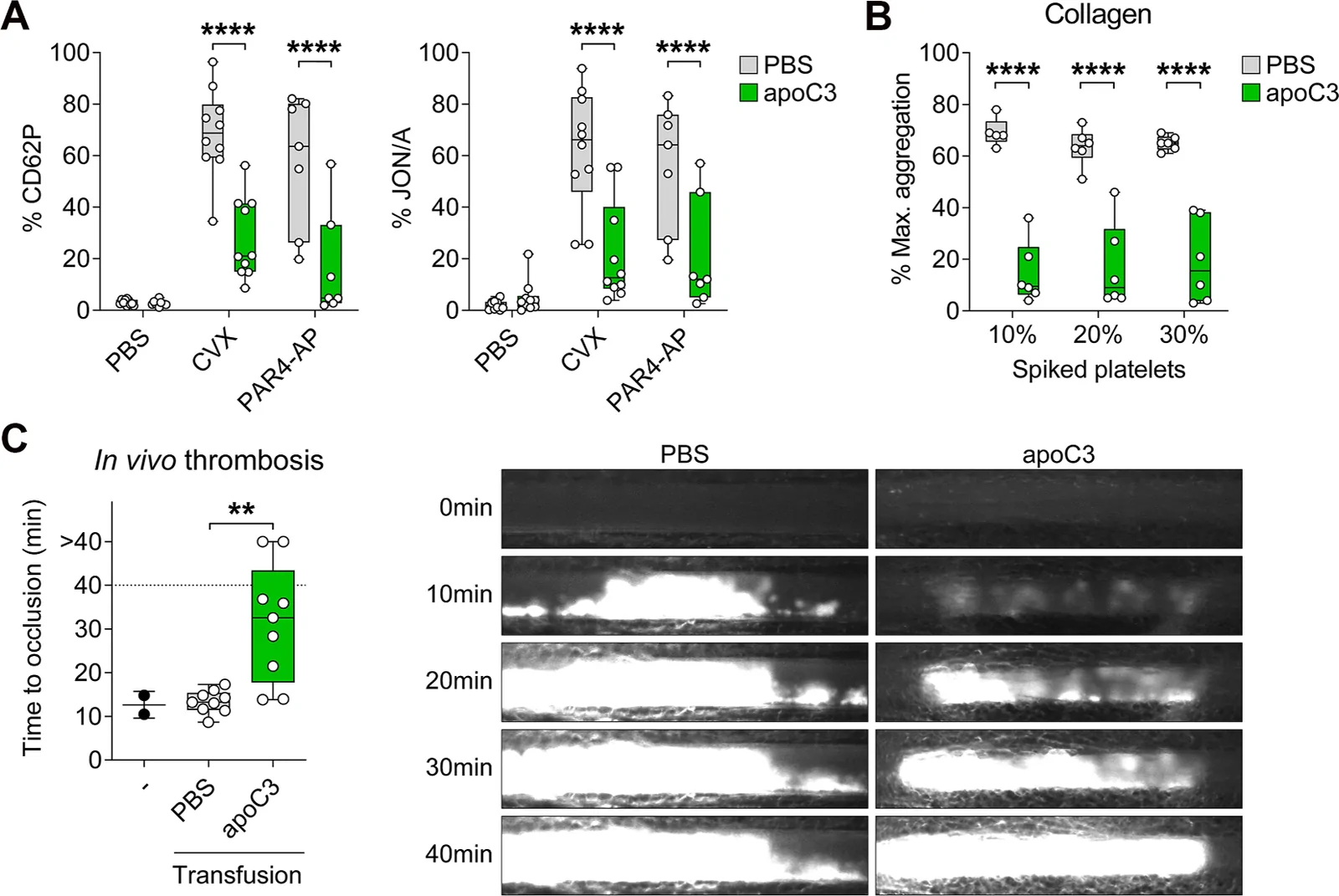
Treatment of platelets with apolipoprotein C3 (apoC3) delays thrombus formation in mice. (A) Washed murine platelets were primed with phosphate-buffered saline (PBS) or 25 μg/mL apoC3 (10 minutes) before stimulation with 200 ng/mL convulxin (CVX) or 25 μM protease-activated receptor 4-activating peptide (PAR4-AP) for 10 minutes. CD62P (left) and α_IIb_β_3_ activation (JON/A antibody binding; right) were quantified by flow cytometry. *n* = 7–10. (B) PBS- or apoC3-primed (25 μg/mL, 10 minutes) isolated murine platelets were spiked into naïve platelet samples at different ratios, yielding a relative fraction of 10% to 30% PBS/apoC3-primed platelets. Platelet aggregation in response to 17 μg/mL collagen was assessed by light transmission aggregometry. *n* = 5–6. (C) Naïve wildtype mice were transfused with 6.9 × 10^8^ donor platelets that had been primed *in vitro* with PBS or 25 μg/mL apoC3 (10 minutes) and subjected to FeCl_3_-induced thrombosis of mesenteric arterioles. Quantification of time to occlusion and representative images showing labeled platelets (white) are shown. *n* = 2 (no transfusion) or *n* = 9 (transfusion) arterioles. **P < .01, ****P < .0001.

Finally, we aimed to shed light on the impact of apoC3 on thrombus formation *in vivo*. To that goal, we first confirmed that apoC3 also inhibited platelet degranulation and integrin activation of isolated murine platelets (Figure 8A). Importantly, we also observed that spiking 10% to 30% apoC3-primed platelets into naïve murine platelets potently reduced aggregation (Figure 8B), demonstrating that treatment of only a minor fraction of platelets was sufficient to impair platelet function.

To investigate the platelet-specific effect of apoC3 on *in vivo* thrombosis, we thus adopted a platelet transfusion approach. We injected naïve wildtype recipient mice with donor platelets that had been primed *in vitro* with PBS or apoC3 (constituting about 25% of all circulating platelets) and subjected them to a model of FeCl_3_-induced thrombosis. Of note, transfusion of control platelets did not expedite thrombus formation, demonstrating that the increased platelet count did not affect physiologic responses. In contrast, transfusion of apoC3-primed platelets significantly delayed time to stable occlusion of mesenteric arterioles (Figure 8C), suggesting that elevated apoC3 levels could indeed modulate thrombus formation *in vivo*. Notably, formed aggregates appeared less stable (Supplementary Video S2), which suggests a role of apoC3 in thromboembolism.

## 4 | DISCUSSION

ApoC3 drives hyperlipidemia and inflammation, making it an important risk factor for ACVD morbidity and mortality. Antisense oligo-nucleotides targeting apoC3 messenger RNA efficiently reduce triglyceride and cholesterol levels, and the first drug of this class has been approved for treatment of familial chylomicronemia syndrome [50,51]. Based on these effects, it has been widely assumed that lowering apoC3 would not only improve plasma lipid profiles but also reduce thrombotic and thromboembolic complications. Our findings challenge this concept, suggesting that the contribution of apoC3 to thrombotic risk is more complex and multifaceted. We identified apoC3 as lipoprotein-derived inhibitor of prothrombotic platelet functions, including degranulation, spreading, and aggregation, that alters AKT and VASP signaling and interferes with actin cytoskeleton remodeling via RGD-containing integrins, thereby expanding current knowledge on the role of apoC3 in vascular disease. Our results demonstrate that despite its proatherogenic properties via lipid metabolism and inflammation, elevated apoC3 may also attenuate thrombotic risk via direct inhibition of platelet activation.

Our study identifies apoC3 as potent platelet inhibitor that profoundly suppresses core prothrombotic responses across different agonists, suggesting interference with central signaling hubs that integrate multiple receptor pathways. Previous studies reported that apoC3 activates monocytes and/or endothelial cells via TLR2/TLR4 and G_i/o_-coupled receptors [26,46], which are also expressed on platelets [32,52]. Moreover, the proteoglycans biglycan and heparan sulfate proteoglycans as well as scavenger receptors SR-B1 and CD36 have been implicated in apoC3 and/or VLDL binding [43–45,47]. However, our results show that among these candidates, only scavenger receptors might play a minor role in apoC3-platelet interactions. Instead, we show that apoC3-mediated platelet inhibition crucially depends on RGD-containing integrins, such as α_5_β_1_ and α_IIb_β_3_, which also convey the antithrombotic effects of apoA4 [48]. Although α_IIb_β_3_-mediated outside-in signaling is classically associated with enhanced platelet activation and aggregation, it can concomitantly trigger negative feedback by activating Src homology 2-containing inositol 5-phosphatase 1 (SHIP1) to terminate PI3K/AKT signaling, restrict degranulation, and inactivate integrins [53].

In platelets, PI3K signals downstream of both G protein-coupled receptors and receptor tyrosine kinases in response to a plethora of agonists, including ADP, thrombin, collagen, and oxLDL, and is pivotal for shape change, degranulation, and aggregation [54–56]. Although apoC3 did not completely abrogate AKT phosphorylation, the reduced PI3K/AKT signaling in apoC3-treated platelets may at least partly explain the functional deficits observed.

Consistent with previous reports that apoC3 binding does not affect gel-to-liquid transition of membrane bilayers [57], we observed no effect of apoC3 on membrane fluidity. ApoC3 also did not affect agonist-induced changes in membrane potential but caused rapid baseline hyperpolarization, which is associated with impaired platelet function [58]. The platelet resting membrane potential of −50 to −60 mV is primarily determined by the voltage-gated K^+^ channel K_v_1.3. After stimulation, platelets further undergo early hyperpolarization via the Ca^2+^-activated K^+^ channel K_Ca_3.1, followed by modest depolarization at later stages due to chloride efflux and cation influx [59,60]. In line with the lack of apoC3 effect on agonist-evoked Ca^2+^ mobilization, our data indicate that apoC3 modulates K_v_1.3 but not K_Ca_3.1 activity. Beyond its ion channel function, K_v_1.3 interacts with β_1_ integrins and modulates α_2_β_1_-mediated platelet adhesion to collagen [59]. This underlines the putative role of the RGDS-sensitive integrin α_5_β_1_ as a platelet apoC3 receptor and as a potential link between apoC3 recognition, hyperpolarization, and functional impairment.

ApoC3-treated platelets also showed decreased spreading, retaining small size and displaying impaired lamellipodia formation, which was accompanied by disrupted actin remodeling and reduced actin network complexity. In line with this result, apoC3 also increased phosphorylation of VASP at S239, which interferes with actin binding and nucleation and thus forestalls filament assembly [61,62]. Lamellipodial actin branching is pivotal for platelet migration by fostering thrombus reorganization and maintaining vascular integrity during inflammation [63,64].

In accordance with the importance of PI3K/AKT and actin dynamics for platelet function, apoC3 impaired stable thrombus formation and delayed vessel occlusion *in vivo*. Of note, this required only a fraction of platelets to be treated with apoC3, suggesting that hyporesponsive platelets may function as “chain breakers,” destabilizing the thrombus. While apoC3 may thus forestall occluding thrombosis at the primary lesion site, it may also foster thromboembolisms at distant sites.

In circulation, apoC3 is mainly transported by VLDL, which is elevated in hyperlipidemia and associated with increased risk for ACVD and cardiovascular events [65,66]. VLDL was previously reported to augment collagen-induced platelet aggregation via CD36 [47]; however, we found that VLDL impaired platelet function to a similar degree as apoC3. Consistently, patients with familial hyperchylomicronemia or familial hypertriglyceridemia display reduced platelet aggregation despite significantly increased VLDL levels [67,68], underscoring the multifaceted regulation of platelet function in lipid disorders. Future studies combining lipid profiling, platelet function testing, and clinical outcomes in patients with and without apoC3 antisense RNA therapy will be crucial to clarify whether apoC3 modulation constitutes antiembolic benefits or poses an un-intended prothrombotic risk.

Platelet dysfunction in hyperlipidemia may also be influenced by other apolipoproteins. The genes encoding apoC3, apoA1, apoA4, and apoA5 cluster on chromosome 11q23, suggesting shared regulation and functional interdependence [69,70]. Although the effects of apoA5 on platelets are still unknown, both apoA1 and apoA4 curtail primary hemostasis either directly via α_IIb_β_3_ or SR-B1 or indirectly by preventing VWF multimerization [48,71,72]. Intriguingly, despite their genomic proximity, these apolipoproteins have diverse and sometimes opposing roles in lipid metabolism and show distinct associations with cardiovascular risk [73–75]. In contrast, apoB100 and apoE activate platelet p38 via the apoB/E receptor and may enhance platelet activation by LDL [76]. Likewise, apoB100-derived apoB100 danger-associated signal 1 and apo(a) prompt platelet hyperreactivity, facilitating degranulation, aggregation, and adhesion [77,78].

Importantly, our experiments used delipidated free apoC3, which represents only a minor fraction of apoC3 *in vivo* [79]. This paucity is likely due to its rapid and efficient binding to phospholipid vesicles, facilitated by its lipophilic and amphipathic nature, curved helices, and semiflexible hinge regions that allow apoC3 to wrap around micelles of variable sizes [39,80]. Accordingly, accumulating evidence suggests that apoC3 displays significant intervesicular mobility as it shuttles between different lipoprotein pools and can also be taken up by extracellular vesicles [81,82]. We and others have shown that apoC3 binding to whole cells seems primarily receptor-driven [26,83]. Free and lipid-bound apoC3 can elicit distinct cellular responses; for example, monocyte inflammasome activation is only evoked by free apoC3 but not lipid-bound apoC3 or VLDL [79]. Notably, in our study, VLDL reproduced the inhibitory effect of apoC3, underlining the physiologic significance of our findings.

Notably, circulating apoC3 exists as multiple proteoforms generated by posttranslational *O*-glycosylation and sialylation, which influence endothelial activation, LPL inhibition, and hepatic clearance. These proteoforms exhibit distinct associations with plasma triglycerides and cardiovascular risk [24,45,84–87]. As we did not characterize proteoform composition of the supplemented apoC3, it remains to be determined whether specific apoC3 proteoforms differentially influence platelet inhibition and thereby modulate thrombotic risk.

Taken together, apoC3 unexpectedly dampens platelet activation by disrupting actin remodeling and modulating AKT and VASP signaling. This identifies apoC3 as a counter-regulator of platelet activation and adds nuance to the dyslipidemia–thrombosis link by revealing a direct inhibitory effect on platelets.

## Supplementary Material

1

2

3

The online version contains supplementary material available at https://doi.org/10.1016/j.jtha.2026.01.004.
