# Supplementary material for: Elevated levels of apolipoprotein C3 impair platelet function and reduce thrombosis
Source: J Thromb Haemost. Author manuscript; Available in PMC 2026 Aug 29. (PMC13525454; doi:10.1016/j.jtha.2026.01.004)
Supplement: Supplementary file 1 [file NIHMS2190670-supplement-3.pdf]

Supplementary Figures

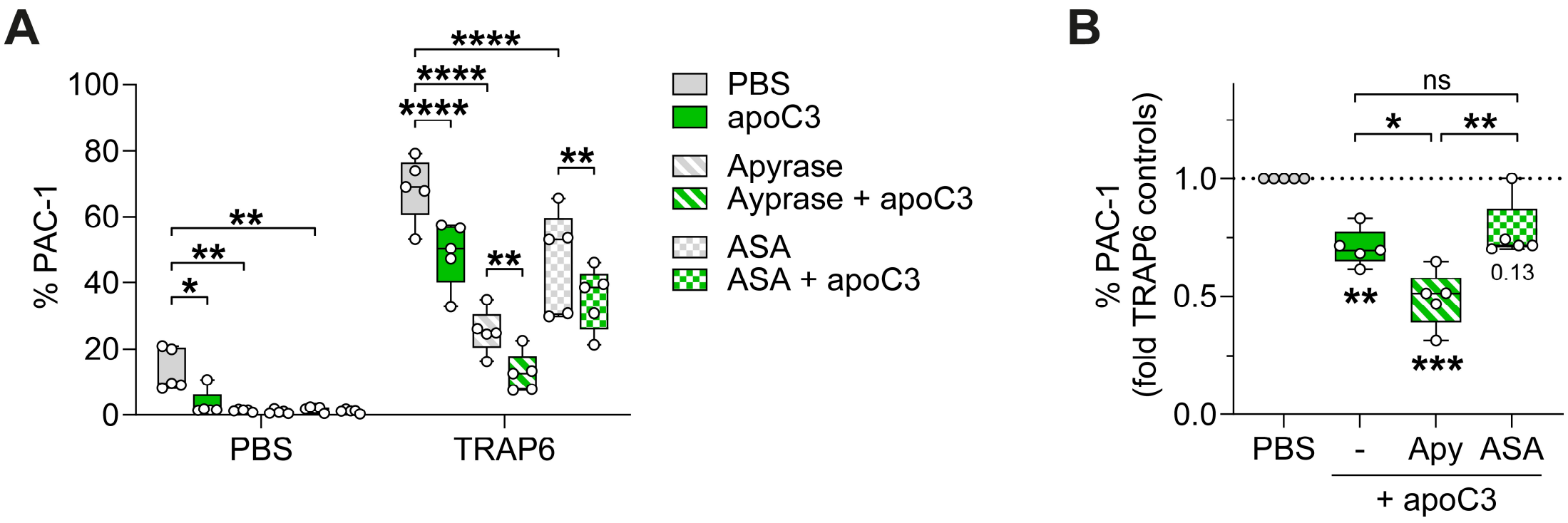

Supplementary Figure S1: ApoC3-mediated platelet inhibition is not mediated by impaired ADP/thromboxane feedback. (A) Washed platelets were pre-treated for 15min with 1U/ml apyrase (Apy) or 1mM acetylsalicylic acid (ASA) before a 10min priming phase with PBS or 25µg/ml apoC3, followed by stimulation with 10µM TRAP6 for 10min. Activation of αIIbβ3 (PAC-1 antibody binding) was quantified by flow cytometry. **(B)** Normalized apoC3 effects in TRAP6-stimulated platelets in dependence of feedback inhibition (Apy or ASA). n=5. *p<0.05, **p<0.01, ***p<0.001, ****p<0.0001. ns: not significant.

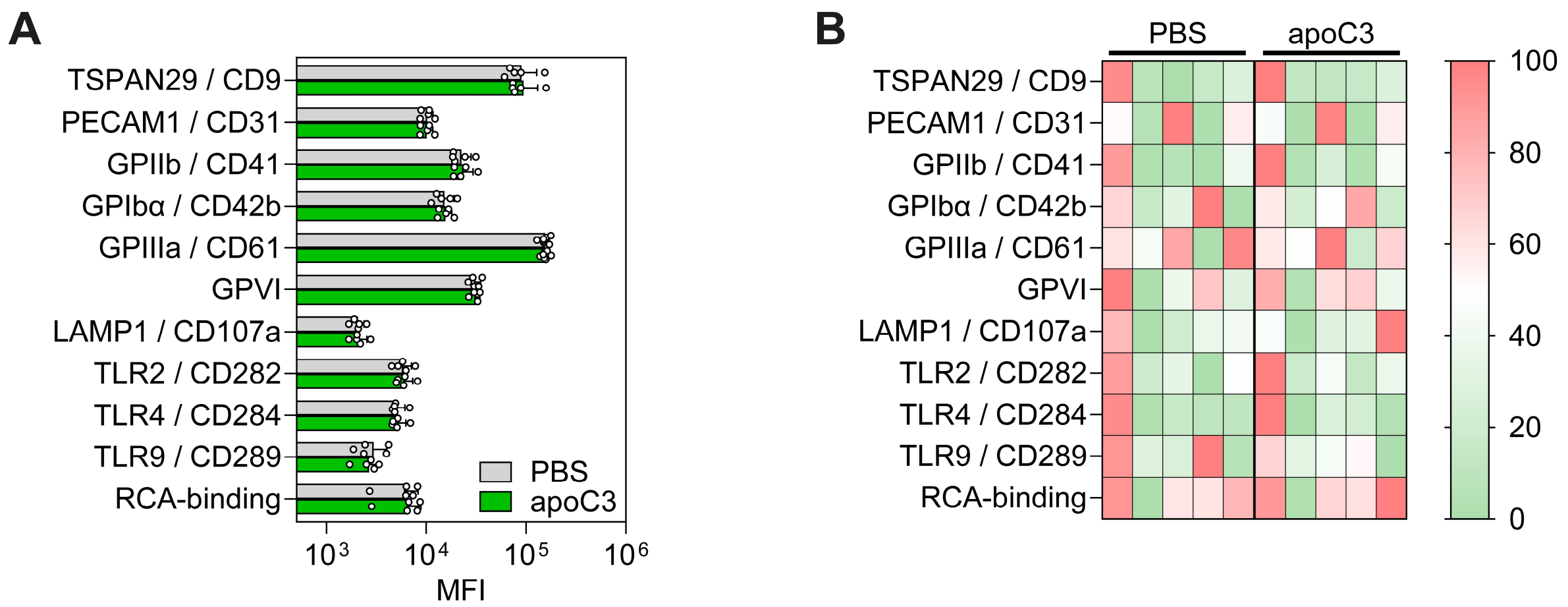

Supplementary Figure S2: ApoC3 does not affect basal surface receptor expression. (A) Platelet-rich plasma was incubated with PBS or apoC3 (50µg/ml, 10min) before surface expression of selected receptors and desialylation (RCA-binding) were detected by flow cytometry. n=5. **(B)** To better visualize relative changes, mean fluorescence intensities (MFI) were normalized to values between 0 (minimum) and 100 (maximum). Heatmap shows individual donors.

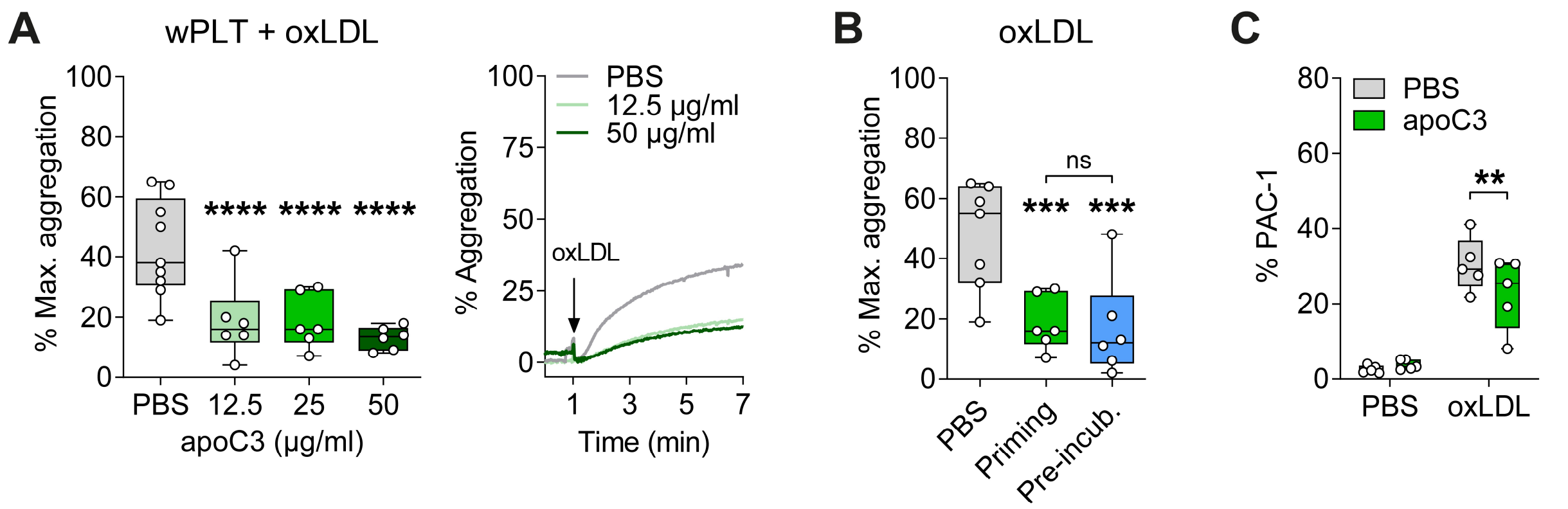

Supplementary Figure S3: ApoC3 impairs oxLDL-induced platelet aggregation. (A) Washed platelets (wPLT) were incubated with PBS or apoC3 (12.5-50µg/ml, 3min) before stimulation with 40µg/ml oxLDL. Platelet aggregation was monitored by light transmission aggregometry (LTA). Representative curve given. n=6-9. **(B)** wPLT were incubated for 3min with PBS or primed with 25µg/ml apoC3 before stimulation with 40µg/ml oxLDL. Alternatively, oxLDL was pre-incubated with apoC3 (3min) before stimulation of wPLT. Platelet aggregation was monitored by LTA. n=6-7. **(C)** wPLT were primed with PBS or 25µg/ml apoC3 (10min) before stimulation with 100µg/ml oxLDL (10min) and quantification of αIIbβ3 activation (PAC-1 antibody binding) by flow cytometry. n=5. **p<0.01, ***p<0.001, ****p<0.0001. ns: not significant.

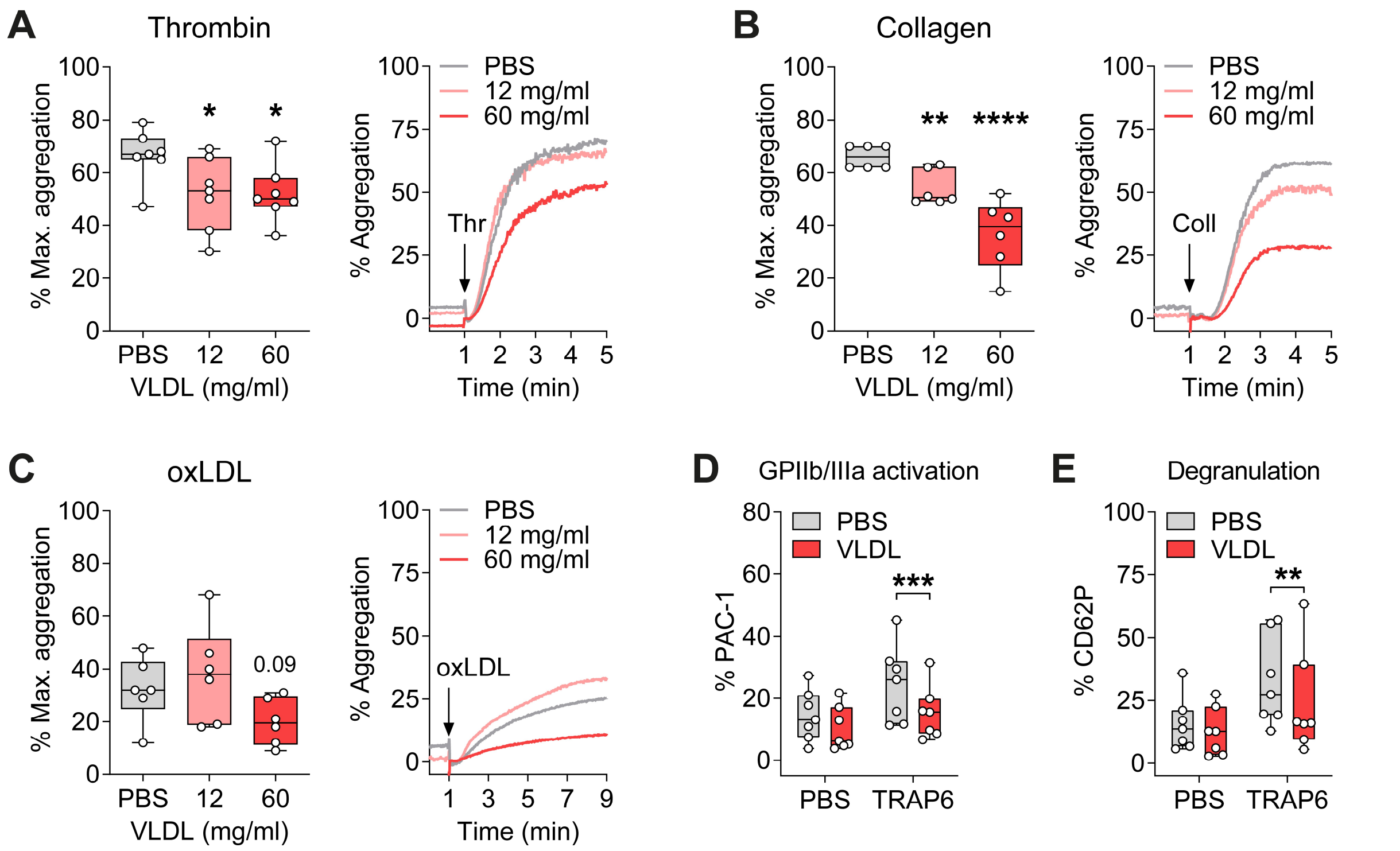

Supplementary Figure S4: VLDL impairs platelet aggregation and activation at hypercholesteremic levels. (A-C) Washed platelets were incubated with PBS or VLDL (12 or 60mg/ml, 3min) before stimulation with (A) 35mU/ml thrombin, (B) 5µg/ml collagen or (C) 40µg/ml oxLDL. Platelet aggregation was monitored by light transmission aggregometry. Representative curves given. n=6-7. **(D-E)** Washed platelets were primed with PBS or 60mg/ml VLDL (10min) before stimulation with 5µM TRAP6 (10min) and quantification of (D) αIIbβ3 activation (PAC-1 antibody binding) or (E) CD62P by flow cytometry. n=7. *p<0.05, **p<0.01, ***p<0.001, ****p<0.0001.

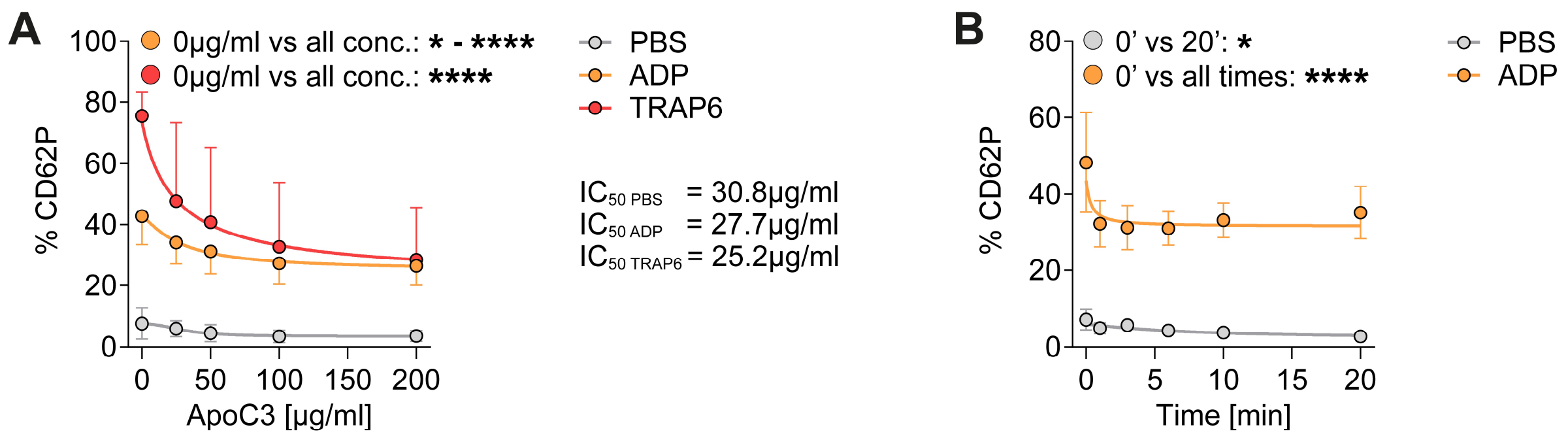

Supplementary Figure S5: ApoC3-mediated platelet inhibition is concentration- but not time-dependent. (A) Washed platelets were primed with PBS or 25-200µg/ml apoC3 (10min) before stimulation with 15µM ADP or 5µM TRAP6 (10min) and quantification of surface CD62P by flow cytometry. n=4. Half maximal inhibitory concentrations (IC_{50}) are indicated. **(B)** Washed platelets were primed with PBS or 25µg/ml apoC3 (1-20min) before stimulation with 15µM ADP (10min) and CD62P quantification. n=3. *$p<0.05$, ****$p<0.0001$.

Supplementary Table S1: Reagents and software

Antibodies				
Flow cytometry				
BV605 anti-human CD45	1:100	HI30	BioLegend	Cat#304042
PE/Cy7 anti-human CD9	1:300	HI9a	BioLegend	Cat#312116
APC anti-human CD11b activated	1:60	CBRM1/5	BioLegend	Cat#301410
AF594 anti-human CD31	1:300	WM59	BioLegend	Cat#303126
PerCP/Cy5.5 anti-human CD36	1:150	5-271	BioLegend	Cat#336224
PE anti-human CD40L	1:50	24-31	BioLegend	Cat#310806
AF700 anti-human CD41	1:200	HIP8	BioLegend	Cat#303728
BV650 anti-human CD42b	1:100	HIP1	BioLegend	Cat#303926
PE anti-human CD61	1:60	VI-PL2	BioLegend	Cat#336406
AF647 anti-human CD61	1:60	VI-PL2	BioLegend	Cat#336408
FITC anti-human CD62L	1:100	DREG-56	BioLegend	Cat#304804
BV605 anti-human CD62P	1:100	AK4	BioLegend	Cat#304906
PB anti-human CD63	1:100	H5C6	BioLegend	Cat#353012
BV650 anti-human CD107a	1:300	H4A3	BioLegend	Cat#328638
PE/Cy7 anti-human CD282 (TLR2)	1:250	TL2.1	BioLegend	Cat#309721
APC/eFluor780 anti-human CD284 (TLR4)	1:200	HTA125	Life Technologies	Cat#47-9917-42
BV421 anti-human CD289 (TLR9)	1:200	S16013D	BioLegend	Cat#394806
FITC anti-human αIIbβ3 activated	1:40	PAC-1	BD Biosciences	Cat#340507
Anti-human phospho-VASP (S239)	1:20	VASP-16C2	nanoTools	Cat#0047-100
FITC anti-mouse IgG	1:40	polyclonal	BD Biosciences	Cat#349031
PE/Cy7 anti-mouse CD62P	1:200	RMP-1	BioLegend	Cat#148310
PE anti-mouse αIIbβ3 activated	1:20	JON/A	Emfret Analytics	Cat#M023-2
Immunofluorescence				
Anti-α/β tubulin	1:100	polyclonal	Cell Signaling	Cat#2148S
DyLight650 anti-rabbit	1:100	polyclonal	Abcam	Cat#ab96922
Western Blot				
Rabbit anti-phospho-AKT (S473)	1:1000	D9E	Cell Signaling	Cat#4060S
Rabbit anti-AKT	1:1000	C67E7	Cell Signaling	Cat#4691S
Rabbit anti-phospho-ERK (Y202/Y204)	1:1000	D13.14.4E	Cell Signaling	Cat#9106S
Rabbit anti-ERK	1:1000	137F5	Cell Signaling	Cat#4695S
Rabbit anti-phospho-p38 (T180/T182)	1:1000	D3F9	Cell Signaling	Cat#4511S
Rabbit anti-p38	1:1000	D13E1	Cell Signaling	Cat#8690S
HRP-linked anti-rabbit IgG	1:2000		Cell Signaling	Cat#7074S
Blocking				
Anti-human CD282 (TLR2)	10µg/ml	TL2.1	BioLegend	Cat#309702
Anti-human CD284 (TLR4)	10µg/ml	HTA125	BioLegend	Cat#312802
Isotype mouse IgG2a, kappa	10µg/ml	MOPC-173	BioLegend	Cat#400202
In vivo				
DyLight488 anti-mouse GPIbβ	5µl/mouse	X488	Emfret Analytics	Cat#X488

Chemicals, Peptides, and Recombinant Proteins		
Apolipoprotein C3	Academy Bio-Medical Company	Cat#33P-105
Convulxin (CVX)	Santa Cruz Biotechnology	Cat#sc-202554
Adenosine diphosphate (ADP)	Sigma-Aldrich	Cat#A2754
Thrombin receptor activator peptide 6 (TRAP6)	AnaSpec	Cat#24190
Protease-activated receptor 4-activating peptide (PAR4-AP; AYPGKF-NH2)	AnaSpec	Cat#AS-60218-1

Collagen I, from calf skin	möLab	Cat#C/N101562
Thrombin reagent	Technoclone	Cat#5100005
Oxidized low-density lipoprotein (oxLDL)	In-house preparation	DOI: 10.1161/ATVBAHA.113.302919
Very low-density lipoprotein (VLDL)	In-house preparation	DOI: 10.1161/01.atv.15.1.17
Apyrase	Sigma Aldrich	Cat#A6535
Acetylsalicylic acid (ASA)	Sigma Aldrich	Cat#A5376
RGDS peptide (Arg-Gly-Asp-Ser)	Tocris Bioscience	Cat#3498
Pertussis toxin (PTX)	List Labs	Cat#181
Maleylated human serum albumin (mHSA)	In-house preparation	DOI: 10.1016/S0049-3848(99)00197-8
Heparin	Tocris Bioscience	Cat#2812
Rhodamine ricinus communis agglutinin (RCA)	Vector laboratories	Cat#VECRL-1082
AF647 bovine lactadherin	CellSystems	Cat#BLAC-ALEXA647
Fluo-4	Thermo Fisher Scientific	Cat#11504786
FuraRed	Thermo Fisher Scientific	Cat#11508966
CellMask Orange	Life Technologies	Cat#10093243
$DiBAC_4(3)$	Fisher Scientific	Cat#B438
Phalloidin-iFluor555 (1:500)	Abcam	Cat#ab176756
Fibronectin	Sigma-Aldrich	Cat#F1141
TNF-α	R&D Systems	Cat#210-TA-005
Prostaglandin I_2 (PGI_2)	Sigma Aldrich	Cat#P6188
SuperSignal West Femto Maximum Sensitivity Substrate	Thermo Fisher Scientific	Cat#34096
Permeabilization buffer	Life Technologies	Cat#00-8333-56
rLuciferase/Luciferin Reagent	Promega	Cat#FF2021
Acid citrate-dextrose (ACD)	Sigma-Aldrich	Cat# C3821-50ML
Heparin	VWR	Cat#BCHRL6510
Ketasol	Livisto	Pharmacy
Xylasol	AniMedica	Pharmacy
**Kits**		
CXCL4/PF4 DuoSet ELISA	R&D Systems	Cat#DY595
CD40 Ligand/TNFSF5 DuoSet ELISA	R&D Systems	Cat#DY617
TSP-1 DuoSet ELISA	R&D Systems	Cat#DY3074
**Experimental Models**		
Human umbilical vein endothelial cells (HUVEC)	In-house isolation	DOI: 10.1161/ATVBAHA.113.302919
C57BL/6 wildtype mice	The Jackson Laboratory	JAX 000664
**Software**		
Graph Pad Prism version 8.0.2 or 10.6.1	GraphPad Software	www.graphpad.com
FlowJo software version 10	Tree Star	www.flowjo.com
CellQuest Pro software	BD Biosciences	www.bdbiosciences.com
C6 Sampler software 1.0.264.21	BD Biosciences	www.bdbiosciences.com
CytExpert version 2.4 and 2.5	Beckman Coulter	www.beckman.de
AlphaEase FC software	Alpha Innotech	genetictechnologiesinc.com/alpha/alpha_ease_fc.htm
Image J 1.52p	DOI: 10.1038/nmeth.2089	imagej.nih.gov/ij
